# Action Potential Firing Patterns Regulate Dopamine Release via Voltage‐Sensitive Dopamine D2 Autoreceptors in Mouse Striatum In Vivo

**DOI:** 10.1002/advs.202412229

**Published:** 2024-12-27

**Authors:** Xiaoxuan Sun, Lili Yin, Zhongjun Qiao, Muhammad Younus, Guoqing Chen, Xi Wu, Jie Li, Xinjiang Kang, Huadong Xu, Li Zhou, Yinglin Li, Min Gao, Xingyu Du, Yuqi Hang, Zhaohan Lin, Liyuan Sun, Qinglong Wang, Ruiying Jiao, Lun Wang, Meiqin Hu, Yuan Wang, Rong Huang, Yiman Li, Qihui Wu, Shujiang Shang, Shu Guo, Qian Lei, Haifeng Shu, Lianghong Zheng, Shirong Wang, Feipeng Zhu, Panli Zuo, Bing Liu, Changhe Wang, Quanfeng Zhang, Zhuan Zhou

**Affiliations:** ^1^ State Key Laboratory of Membrane Biology National Biomedical Imaging Center and Institute of Molecular Medicine College of Future Technology Peking‐Tsinghua Center for Life Sciences PKU‐IDG/McGovern Institute for Brain Research Peking University Beijing 100871 China; ^2^ Peking University Sixth Hospital Peking University Institute of Mental Health NHC Key Laboratory of Mental Health (Peking University) National Clinical Research Center for Mental Disorders (Peking University Sixth Hospital) Beijing 100191 China; ^3^ Neuroscience Research Center Institute of Mitochondrial Biology and Medicine Key Laboratory of Biomedical Information Engineering of Ministry of Education School of Life Science and Technology Xi'an Jiaotong University Xi'an 710049 China; ^4^ Department of Neurology the Second Affiliated Hospital of Xi'an Jiaotong University Xi'an 710004 China; ^5^ Key Laboratory of Medical Electrophysiology Ministry of Education of China Collaborative Innovation Center for Prevention and Treatment of Cardiovascular Disease and the Institute of Cardiovascular Research Southwest Medical University Luzhou 646000 China

**Keywords:** action potential, auto‐inhibition, dopamine receptor 2, dopamine release in vivo, firing pattern

## Abstract

Dopamine (DA) in the striatum is vital for motor and cognitive behaviors. Midbrain dopaminergic neurons generate both tonic and phasic action potential (AP) firing patterns in behavior mice. Besides AP numbers, whether and how different AP firing patterns per se modulate DA release remain largely unknown. Here by using in vivo and ex vivo models, it is shown that the AP frequency per se modulates DA release through the D2 receptor (D2R), which contributes up to 50% of total DA release. D2R has a voltage‐sensing site at D131 and can be deactivated in a frequency‐dependent manner by membrane depolarization. This voltage‐dependent D2R inhibition of DA release is mediated via the facilitation of voltage‐gated Ca^2+^ channels (VGCCs). Collectively, this work establishes a novel mechanism that APs per se modulate DA overflow by disinhibiting the voltage‐sensitive autoreceptor D2R and thus the facilitation of VGCCs, providing a pivotal pathway and insight into mammalian DA‐dependent functions in vivo.

## Graphical abstract

Sun et al. uncover a novel dopamine transmission pathway: the membrane potential or action potential firings not only trigger dopamine release, but also modulate dopamine release by directly targeting voltage‐sensitive D2 receptor via the voltage‐sensing site D131, mediating the facilitation of voltage‐gated calcium channels, thus tuning dopamine release in mammalian brain in vivo.

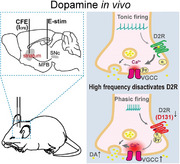

## 1 Introduction

Dopamine (DA) is essential in the modulation of movement, motivation, learning, and reward.^[^ ^1^ ^]^ Dysfunctions of dopaminergic homeostasis are causally linked to Parkinson's disease, schizophrenia, and addiction.^[^ ^2^ ^]^ Midbrain DA neurons in the substantia nigra pars compacta projecting to the dorsal striatum via the medial forebrain bundle (MFB) have been extensively studied due to their central roles in motor control and in the pathology of Parkinson's disease.^[^ ^2^, ^3^ ^]^ Midbrain DA neurons display a range of activity states, from low‐frequency sustained (tonic) firing to bursts of high‐frequency (phasic) action potentials (APs).^[^ ^4^ ^]^ Tonic DA release establishes a basal background DA level and phasic DA release provides transient rise and fall of DA levels, which is thought to drive movement initiation and reward‐based learning.^[^ ^5^ ^]^ To investigate the influence of these different AP firing patterns or frequencies on DA release, extracellular DA has been electrochemically monitored in real time by using a micro carbon fiber electrode (CFE) in striatal slices and in vivo.^[^ ^4^, ^6^ ^]^

AP firing triggers DA release from presynaptic terminals, while DA mediates its effects by binding to two major classes of G‐protein‐coupled receptors (GPCRs): D1‐like Gs‐coupled excitatory receptors (D1 and D5) and D2‐like Gi/o‐coupled inhibitory receptors (D2, D3, and D4).^[^ ^7^ ^]^ D2R has two alternative splicing isoforms, D2 short (D2S) and D2 long (D2L), in which D2S is expressed in presynaptic DA terminals as an auto‐inhibitory receptor while D2L is highly expressed in postsynaptic medium spiny neurons mediating downstream signaling pathways.^[^ ^8^ ^]^ Activation of the D2R autoreceptor provides negative feedback on presynaptic DA release by modulating the firing patterns of DA neurons via D2R‐Giβγ‐mediated inhibition of voltage‐gated Ca^2+^ channels (VGCCs) or activation of G protein‐gated inwardly‐rectifying K^+^ (GIRK) channels.^[^ ^9^ ^]^ However, whether and how DA release with different AP patterns is differentially modulated by D2R remain virtually unknown.

Some GPCRs, such as the muscarinic M2,^[^ ^10^ ^]^ adrenergic α2A,^[^ ^11^ ^]^ and purinergic P2Y_1_^[^ ^12^ ^]^ receptors, have been proposed to be sensitive to the membrane potential (Vm) and thus modulate the downstream signal transduction. Most of the evidence relies on ionic currents through the gating current, GPCR downstream GIRK current, or FRET‐based reporters in reconstituted system in vitro.^[^ ^13^ ^]^ Recently, more physiological evidences for GPCR Vm‐dependence have been reported: the Vm‐dependence of purinergic receptor P2Y_12_ regulates vesicular quantal catecholamine release in mammalian adrenal chromaffin cells (ACCs) by our lab^[^ ^14^ ^]^ and the Vm‐dependence of muscarinic type A receptor controls neuronal plasticity in drosophila by Parnas group.^[^ ^15^ ^]^ However, it is still unclear whether GPCRs can be regulated by neuronal excitability and contribute to neurotransmitter release during synaptic transmission in the central nervous system.

Here, by using electrochemical recording of DA release in vivo in intact animals and ex vivo in striatal slices, introducing three reconstituted D2R activation reporting bioassays, and combining pharmacology, D2R‐knockout (KO) mice, and molecular mutations, we revealed that AP firing pattern D2R‐dependently modulates DA release in situ and in vivo in the striatum. D2R is sensitive to voltage mainly via the D131 site that is in the conserved DRY motif of class A GPCRs. Crucially, high‐frequency AP firings counteract the auto‐inhibitory effect of D2R on DA release via the facilitation of VGCCs. This noncanonical mechanism may provide novel insight into D2R/DA‐related behaviors and pathogenic mechanisms.

## 2 Results

### 2.1 D2R AP Frequency‐Dependently Modulates DA Release In Vivo

**Figure 1:**
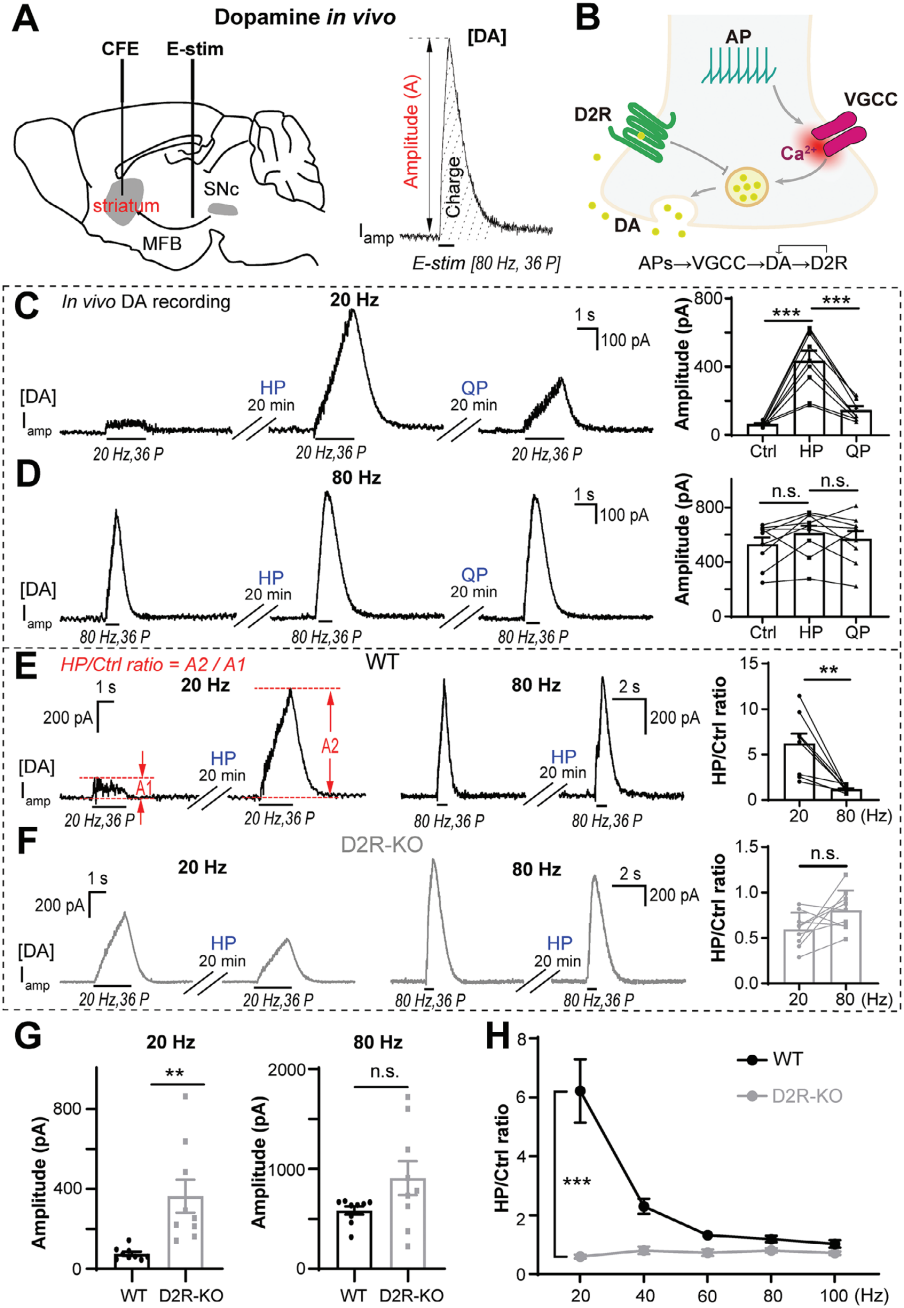
Modulation of dopamine release by GPCR‐D2R autoreceptor is dependent on the frequency of action potentials in mouse striatum in vivo. A) Diagram of the experimental setup (left) of real‐time recording of action potential (AP)‐induced DA overflow [DA] (right) in a mouse striatum in vivo (see the Experimental Section). Right, a typical amperometric recording (*I*_amp_) of [DA] following 36 E‐stim pulses at 80 Hz at the MFB. The amplitude and charge are defined to quantify the [DA]. B) DA transmission model in a striatal terminal. DA release is evoked by Ca^2+^ influx through voltage‐gated Ca^2+^ channels (VGCCs) opened by the APs. The released DA in turn targets the presynaptic D2R auto‐receptor to regulate DA release. C,D) Pharmacology in WT‐mice, with the low (C, [20 Hz, 36 P]) or high (D, [80 Hz, 36 P]) frequency AP‐pattern stimuli at the MFB by the D2R‐antagonist haloperidol (HP, 0.4 mg kg^−1^, *i.p*.) and subsequent D2R‐agonist quinpirole (QP, 8 mg kg^−1^, *i.p*.) [One‐way ANOVA, post hoc Tukey's multiple comparisons test; (C) and (D) were from the same nine mice]. E,F) Typical traces and statistics of E‐stim (20 Hz vs 80 Hz)‐evoked DA release in WT or D2R‐KO mice pre‐ and post‐HP treatment [paired Student's *t*‐test for (E) and (F), *n* = 9 for each group]. G) Statistics of DA release in WT and D2R‐KO mice show significant difference at Estim 20 Hz but not 80 Hz (Welch's *t*‐test, *n* = 9 for each group). H) AP frequency‐dependence of [DA] ratio (HP/Ctrl) in WT versus D2R‐KO mice at different frequencies. Increasing AP frequency from 20 to 100 Hz gradually reduces HP/Ctrl in WT mice (black line), but not in D2R‐KO mice (gray line), indicating that (1) the effect of HP on [DA] is via D2R; (2) the D2R modulation of [DA] depends on AP frequency in vivo (two‐way ANOVA test; *n* = 9 for each group). Data are presented as the mean ± SEM. **p < 0.01, ***p < 0.001; *p* > 0.05, n.s., not significant.

To record the activity‐induced DA overflow [DA] in vivo, a bipolar electrode was inserted into the MFB to elicit electric stimulation (E‐stim) and the AP‐evoked DA release in the striatum was recorded as an amperometric current (*I*_amp_) using a CFE with the holding potential at +780 mV.^[^ ^6^, ^16^ ^]^ With fast‐scan cyclic voltammetry (FSCV) recording,^[^ ^17^ ^]^ we have confirmed that the amperometric currents in the mouse striatum following E‐stim at the MFB represents DA release in vivo.^[^ ^6^ ^]^ When a 36‐pulse burst of E‐stim was applied to the MFB, the typical [DA] signal with two parameters of amplitude (pA) and total charge (pC) were recorded with a CFE to quantify DA release (**Figure** **1**A,B). By using in vivo DA recording in intact C57 mice, we found that the DA release evoked by a burst stimulation using a train of 36 pulses at 20 Hz [20 Hz, 36 pulses] was increased greatly by the D2R antagonist haloperidol,^[^ ^18^ ^]^ which was re‐inhibited by the D2R agonist quinpirole (QP) (Figure 1C). In contrast, HP and QP had almost no detectable effect on DA release evoked by 80 Hz E‐stim (Figure 1D). Importantly, the effects of HP on the amplitude and charge of [DA] were gradually attenuated along with the increase of E‐stim frequency from 20 to 100 Hz (Figure S1, Supporting Information). The voltage‐sensitive modulation of D2R on DA release also occurs under physiological conditions when compared low (10 Hz) versus high (40 Hz) frequency of stimulation (Figure S2, Supporting Information). Consistent with this, raclopride (RP), another D2R antagonist, increased the evoked [DA] signal only at 20 Hz, but not at 80 Hz (Figure S3, Supporting Information). Meanwhile, D2R agonist quinpirole (QP) also displayed frequency‐dependent inhibition on [DA] (Figure S4, Supporting Information). The invalidated function of HP in enhancing DA release upon 80 Hz E‐stim was not attributed to the depletion of releasable vesicle pool because the evoked [DA] signal was further increased when the E‐stim pulses increased from [80 Hz, 36 pulses] to [80 Hz, 72 pulses] (Figure S5, Supporting Information). These data suggest an interesting phenomenon that D2R modulation of the [DA] signal is AP frequency‐dependent in vivo.

We then introduced D2R‐knock out (KO) mice to validate the AP frequency‐dependent modulation of DA release by D2R. Consistent with the pharmacological results (Figure 1C,D), HP specifically increased the E‐stim evoked [DA] at 20 Hz but not 80 Hz (Figure 1E) in WT mice, while in D2R‐KO mice, HP had no effect on the evoked [DA] signal at either 20 or 80 Hz (Figure 1F). The effect of HP on [DA] was quantified as the amplitude ratio of [DA] after/before HP treatment (HP/Control), which was much larger at 20 Hz than that at 80 Hz in WT mice (Figure 1E), but not in D2R‐KO mice (Figure 1F). The [DA] in WT mice was much smaller than that of D2R‐KO mice when stimulated with APs at 20 Hz but not 80 Hz (Figure 1G). The HP/Ctrl ratios evoked by AP frequencies ranging from 20 to 100 Hz gradually decreased, suggesting that the effect of HP on [DA] was frequency‐dependent in WT mice, and this effect was abolished in D2R‐KO mice (Figure 1H). Consistent with this, the [DA] evoked by different AP frequencies ranging from 20 to 80 Hz showed greater frequency‐dependence in WT mice than in D2R‐KO mice (Figure S6, Supporting Information). As a negative control, the modulatory effect of the DA transporter (DAT) antagonist nomifensine on [DA] showed no AP frequency‐dependence (Figure S7, Supporting Information), suggesting the insensitivity of DAT to AP frequency. Consistent with this, the frequency‐dependent effect of D2R antagonist HP on [DA] remained unchanged in the presence of nomifensine (Figure S8, Supporting Information). Combining the results from D2R pharmacology and D2R‐KO mice, we conclude that D2R modulation of [DA] is dependent on AP frequency in vivo.

### 2.2 D2R AP Frequency‐Dependently Modulates DA Release in Striatal Slices In Situ

**Figure 2:**
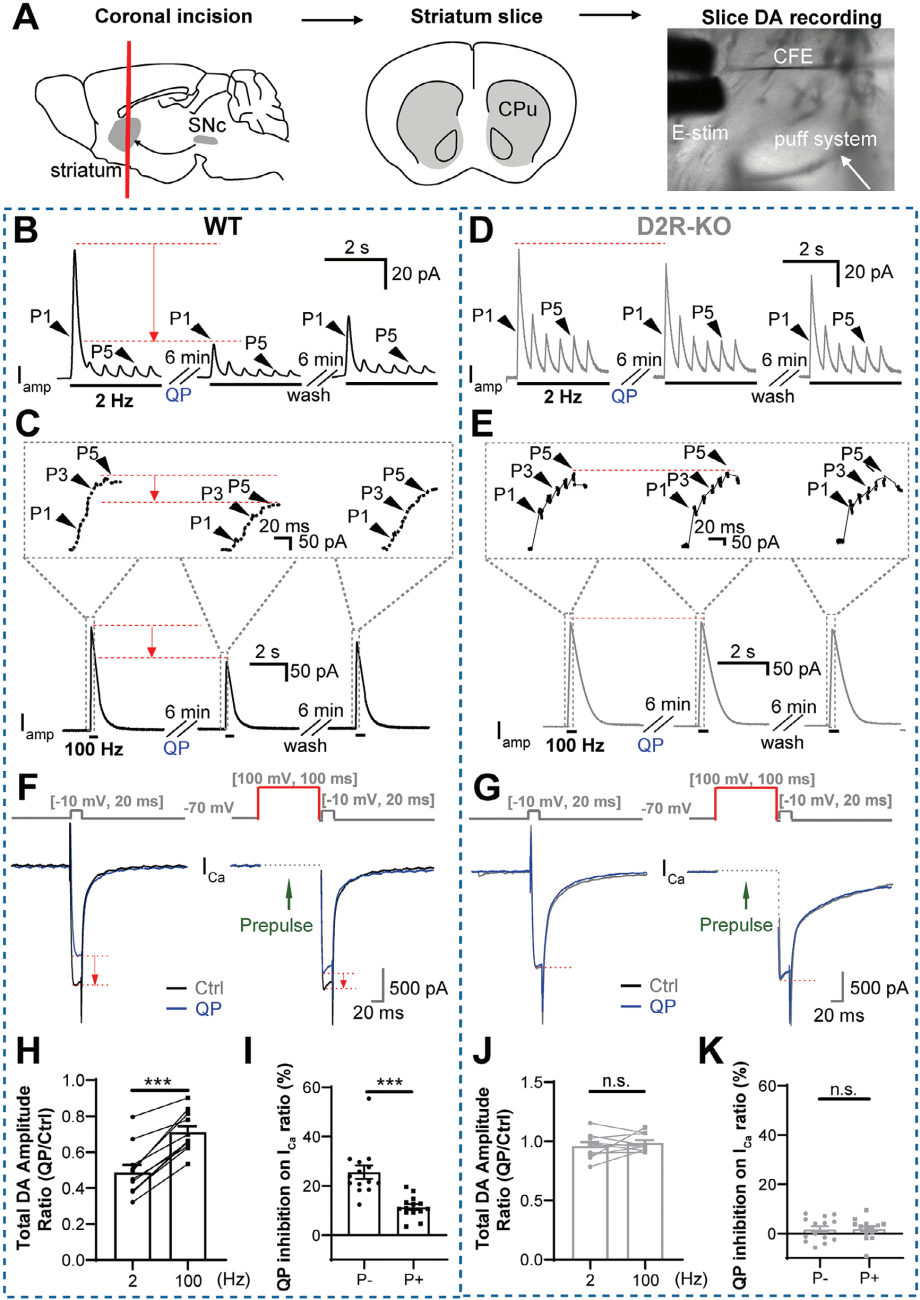
The modulation of AP‐evoked DA release and *I*_Ca_ are frequency‐dependent in WT but not D2R‐KO striatal slices. A) Diagram of real‐time DA recording in striatal slice. Coronary slices (300‐µm thick) were cut from the caudate putamen (CPu) or striatum region of the mouse brain and are bathed in 1 × 10^−6^ m DHβE to block the CTDT pathway (for details see Figure S9, Supporting Information). A stimulating electrode is used to generate different AP patterns and trigger DA release. The evoked DA signals are recorded by the CFE (200‐µm long). Drugs are delivered to the slices via a puffing system. B,C) In WT mice, representative traces of evoked DA release before and after D2R agonist QP (0.5 × 10^−6^ m) treatment with E‐stim patterns of 6 pulses at 2 Hz versus 6 pulses at 100 Hz. The enlarged insets show details of DA signals by 6 pulses (P1–P6) of E‐stim. D,E) Same as (B) and (C) but in D2R‐KO mice. F,G) Typical traces of QP inhibition on *I*_Ca_ with or without a prepulse depolarization effect in WT and D2R‐KO DA neurons. H) Statistics of (B) and (C) showing the effect of QP on total DA release (sum of *I*_P1_ + *I*_P2_ +…+ *I*_P6_) at two E‐stim frequencies: [2 Hz 6 P] versus [100 Hz 6 P] (paired Student's *t*‐test, ***p < 0.001, *n* = 11 slices from 5 mice). I) Statistics showing that a prepulse depolarization (100 mV, 100 ms) mostly removed the blockade effect of QP on *I*_Ca_ in WT DA neurons (QP inhibition on *I*_Ca_ ratio, P−: 25.54% ± 2.70% vs P+: 11.36% ± 1.18%, paired Student's *t*‐test, *p* < 0.001, *n* = 14 cells). J) Statistics of (D) and (E). QP does not alter DA release at both AP frequencies, indicating that the AP frequency‐dependence of the D2R modulation of DA release is abolished in D2R‐KO slices (paired Student's *t*‐test, *p* = 0.31, n.s., not significant, *n* = 10 slices from 4 mice). K) Statistics showed no effect of prepulse depolarization on *I*_Ca_ in D2R‐KO DA neurons (QP inhibition on *I*_Ca_ ratio, P−: 1.64% ± 1.28% vs P+: 1.78% ± 1.16%, paired Student's *t*‐test, *p* = 0.9120, *n* = 14 cells). Data are presented as the mean ± SEM. ***p < 0.001; *p* > 0.05, n.s., not significant.

Considering that DA recording in vivo is complex and involves multiple neural pathways, to confirm the AP frequency‐dependent DA release, we prepared fresh coronal striatal slices. 300‐µm‐thick sections were cut through the striatum, and a bipolar stimulating electrode was placed to elicit DA release in situ. A CFE was inserted into the slice to record DA release, and a drug puff system was used for local drug delivery (**Figure** **2**A).^[^ ^6^, ^19^ ^]^ Since it has been reported that E‐stim can also simultaneously elicit cholinergic interneurons to trigger the second phase of DA release, we used pretreatment with 1 × 10^−6^ m dihydro‐β‐erythroidine hydrobromide (DHβE) to block this cholinergic transmission‐induced DA transmission (CTDT) pathway^[^ ^6^, ^20^ ^]^ and record direct E‐stim‐evoked DA release (Figure S9, Supporting Information). A burst of 6 pulses at 2 or 100 Hz was applied to trigger DA release and the D2R agonist QP was used to test the D2R modulation of DA release. By comparing the total DA amplitude following 6 pulses, the DA release decreased 53% after QP treatment at 2 Hz, while only 31% reduction was obtained upon 100 Hz E‐stim (Figure 2B,C,H), suggesting that the regulation of DA release by QP was weakened by a high AP frequency. Notably, the QP modulation of DA release was reversible, which made up the shortcomings of in vivo recording. Meanwhile, the frequency‐dependence of the QP regulation of DA release was abolished in D2R‐KO mice (Figure 2D,E,J). Together, these results suggest that the D2R modulation of DA release is AP frequency‐dependent in striatal slices in situ.

We next assessed whether the AP frequency‐dependent D2R effect on DA release is mediated via the facilitation of voltage‐gated Ca^2+^ channels (VGCCs).^[^ ^21^ ^]^ Local application of D2R agonist QP greatly inhibited the depolarization (−10 mV, 20 ms)‐evoked *I*_Ca_ in WT‐DA neurons (Figure 2F,I, left, QP inhibition on *I*_Ca_: 25.54% ± 2.70%), which was abolished in that from D2R‐KO mice (Figure 2G,K, left, QP inhibition on *I*_Ca_: 1.64%±1.28%), confirming the inhibitory role of D2R on VGCC activation as reported previously.^[^ ^22^ ^]^ Interestingly, we further found that a prepulse depolarization (100 mV, 100 ms; mimicking the high frequency/phasic APs firing) substantially reduced the blockade effect of QP on *I*_Ca_ (Figure 2F,I, right; QP inhibition on *I*_Ca_: 11.36%±1.18%), which was absent in D2R‐KO DA neurons (Figure 2G,K, right; QP inhibition on *I*_Ca_: 1.78%±1.16%). Thus, D2R's inhibitory effect on *I*_Ca_ is, in addition to VGCC, also Vm‐dependent. Importantly, application of the L‐type calcium channel (LTCC) blocker nifedipine completely abolished the AP frequency‐dependent modulation of DA release by D2R agonist QP (Figure S10, Supporting Information). Together, these results suggest that LTCCs contribute to the AP frequency‐dependent modulation of DA release via D2R.

### 2.3 The Voltage‐Dependent Modulation of D2R Is Validated in Reconstituted ACCs

Thus, the modulation of DA overflow by D2R was AP frequency‐dependent, as validated by D2R‐related pharmacological (agonist QP and antagonists HP/RP) and genetic (D2R‐KO) evidences in vivo (Figure 1 and Figures S1–S8, Supporting Information) and ex vivo (Figure 2 and Figures S9–S10, Supporting Information). To further investigate the mechanism, we used rat adrenal chromaffin cells (ACCs), a classic neuroendocrine model for the study of stimulation‐secretion coupling.^[^ ^23^ ^]^ Previously, we found that quantal neurotransmitter release from a single vesicle, i.e., quantal size (QS), can be inhibited by the activation of Gi‐coupled GPCRs‐somatostatin receptor^[^ ^24^ ^]^ and P2Y_12_ receptor^[^ ^14^ ^]^ via Giβγ‐mediated limitation of vesicle fusion pore expansion, resulting in a smaller QS in ACCs.^[^ ^24^, ^25^ ^]^ Thus, in principle, the QS change induced by Gi‐GPCR activation can be used as a “GPCR‐ACC‐QS bioassay” to report the function of Gi‐GPCRs in ACCs. Immunostaining showed that native rat ACCs did not express endogenous D2R (Figure S11A,B, Supporting Information), permitting ACCs to report D2R function by overexpressing exogenous D2R (the short isoform D2S, which is specifically expressed in presynaptic DA neurons, ACC‐D2R). Unlike GFP‐overexpressing control cells (ACC‐Ctrl, Figure S11C, Supporting Information), the D2R agonist QP significantly inhibited the QS of catecholamine release evoked by caffeine (a ryanodine receptor activator) in reconstituted ACC‐D2R cells (Figure S11D, Supporting Information) mostly via the GPCR‐Giβγ‐vesicle fusion pore pathway.^[^ ^14^, ^24^ ^]^ The reconstituted D2R‐ACCs‐QS bioassay provided a powerful D2R activation reporting system via the regulation of quantal vesicular release.

**Figure 3:**
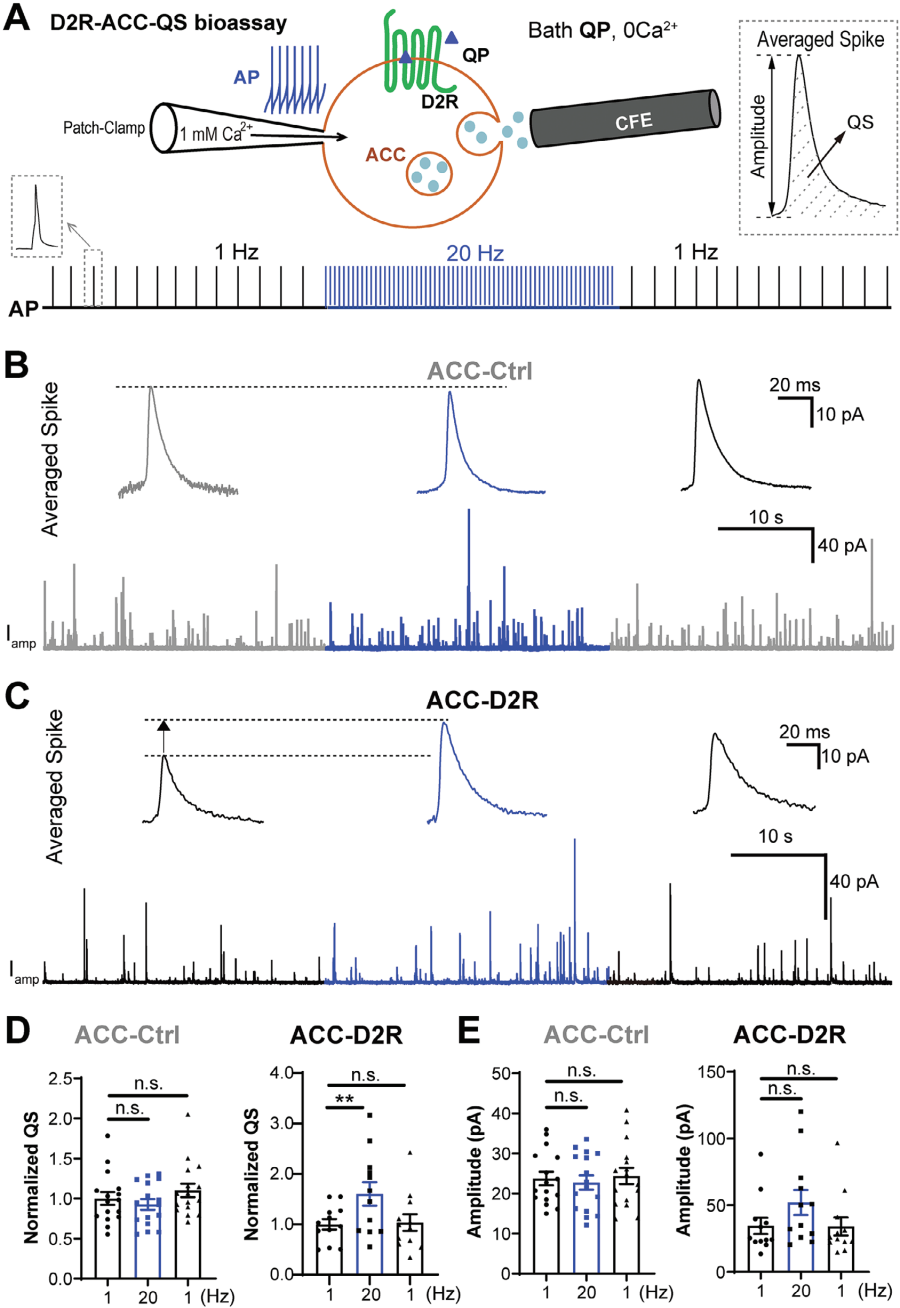
The voltage dependence of D2R is validated in native secretary adrenal chromaffin cells (ACCs) overexpressing D2R. A) Diagram of combined patch‐clamp and CFE recordings in an ACC over‐expressing D2R. ACCs were bathed in 2 × 10^−6^ m QP and 0 Ca^2+^ extracellular solution, and whole‐cell dialysis with 1 × 10^−3^ m intracellular Ca^2+^ in the patch pipette was used to trigger quantal release, which can be real‐time recorded by a CFE as *I*_amp_ current. Simultaneously, through the whole‐cell patch‐clamp, two AP frequencies (1 Hz vs 20 Hz) stimulus were used to depolarize the ACCs during quantal vesicular release. Dashed boxes show a single AP waveform (left) and an averaged quantal spike signal (right), respectively. B) A representative amperometric recording of vesicle quantal release (*I*_amp_, lower trace) evoked by the stimulation protocol shown in panel A in ACCs transfected with GFP control plasmid (ACC‐Ctrl). Insets show the averaged quantal size (QS) corresponding to three periods of stimulation at [1 Hz 30 s] (gray) versus [20 Hz 30 s] (blue). C) Same as (B) but in ACCs transfected with D2R plasmid (ACC‐D2R). D) Statistics of normalized QS. For ACC‐Ctrl, statistics show that QS does not change significantly during 1 Hz versus 20 Hz stimulation. For ACC‐D2R, statistics show that high frequency (20 Hz) significantly increases QS compared to low frequency (1 Hz) (one‐way ANOVA test, post hoc Tukey's multiple comparisons test, *p* > 0.05, n.s.; **p < 0.01, *n* = 16 cells for ACC‐Ctrl and *n* = 12 cells for ACC‐D2R). e) Statistics of the quantal release amplitude during 1 Hz versus 20 Hz stimulation in ACCs transfected with control (ACC‐Ctrl) or D2R‐expressing (ACC‐D2R) plasmid. (One‐way ANOVA, post hoc Tukey's multiple comparisons test, *p* > 0.05, n.s., not significant, *n* = 16 cells for ACC‐Ctrl and *n* = 12 cells for ACC‐D2R). Data are presented as the mean ± SEM. **p < 0.01, ***p < 0.001; *p* > 0.05, n.s., not significant.

Based on the D2R‐ACCs‐QS reporting system, we used combined whole‐cell patch‐clamp and CFE recording to confirm the AP frequency‐dependent modulation of D2R and vesicular secretion. ACCs were bathed in 2 × 10^−6^ m QP‐containing and Ca^2+^‐free extracellular solution (to exclude the impact of Ca^2+^ influx on secretion during membrane depolarization), then 1 × 10^−3^ m Ca^2+^‐containing intracellular solution was used to trigger vesicular release from a patched cell, and stimulation at different AP frequencies (1 Hz vs 20 Hz) was applied via patch‐clamp to depolarize the ACCs (**Figure** **3**A). We found that the QS of catecholamine release^[^ ^24^ ^]^ remained unchanged between 1 and 20 Hz in ACC‐Ctrl cells (Figure 3B,D). Strikingly, in ACC‐D2R cells, 20 Hz stimulation significantly increased the QS compared with that at 1 Hz (Figure 3C,D). Similarly, pulse depolarization from −70 to 0 mV significantly increased the QS in ACC‐D2R but not ACC‐Ctrl cells (Figure S12A–D, Supporting Information). The amplitude of catecholamine quantal release also displayed increase trends (*p* = 0.0607 for 1 vs 20 Hz) while with no significance (Figure 3E and Figure S12E, Supporting Information). In contrast, the frequency of quantal catecholamine release remained unchanged following high‐frequency stimulation or membrane depolarization (Figure S12F,G, Supporting Information). Nonetheless, these data demonstrate that both high AP frequency and step‐depolarization removed the D2R inhibition of vesicular quantal release in reconstituted ACCs, suggesting that the modulation by D2R is sensitive to membrane voltage (Vm). Similar to that of whole‐cell Ca^2+^ dialysis, when caffeine was used to evoke catecholamine release, the voltage‐dependent modulation of D2R on quantal secretion was reproduced (Figure S11E,F, Supporting Information), supporting the voltage‐dependent deactivation of D2R in reconstituted ACC‐D2R cells.

### 2.4 D2R Vm‐Sensing Site D131 Is Revealed in Reconstituted GIRK System

**Figure 4:**
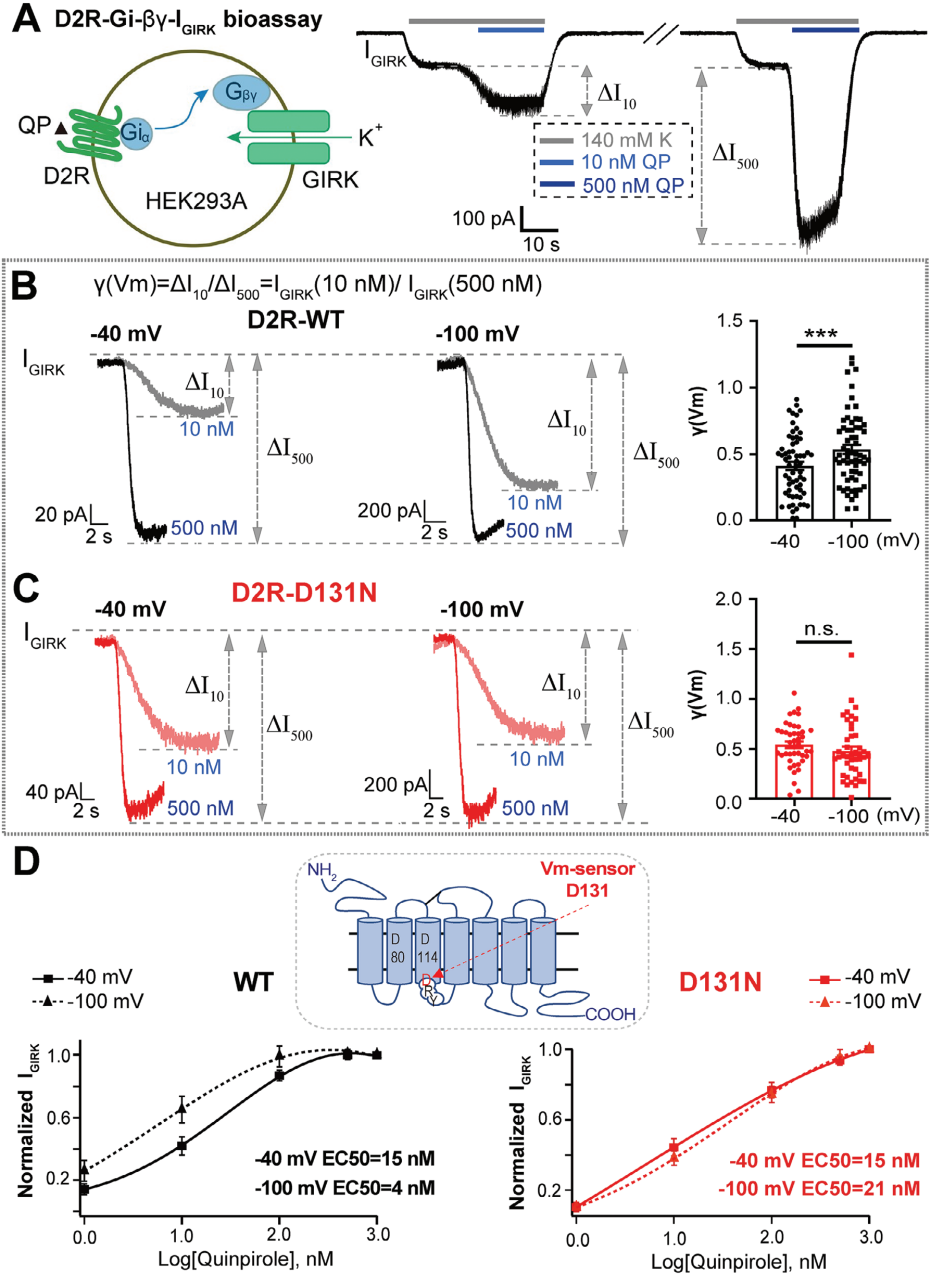
A D2R voltage‐sensing site (D131) as revealed with a reconstituted GIRK system overexpressing D2R. A) Left, cartoon illustration of the D2R‐G_i_‐βγ‐GIRK current assay. GIRK1/4 and D2R plasmids are co‐expressed in HEK293A cells, and the D2R agonist QP activates the D2R‐Gi pathway to induce GIRK current (*I*_GIRK_) in 140 × 10^−3^ m KCl (140K) solution. Right, Whole‐cell recording of *I*_GIRK_ following 10 and 500 × 10^−9^ m QP treatment (Δ*I*_10_ and Δ*I*_500_ are *I*_GIRK_ induced by 10 and 500 × 10^−9^ m QP). Note, *I*_GIRK_ is larger at 500 × 10^−9^ m that at 10 × 10^−9^ m QP. B) Left, *I*_GIRK_ evoked by 10 or 500 × 10^−9^ m QP measured at a holding Vm of −40 or −100 mV in cells co‐expressing D2R‐WT and GIRK1/4. The *I*_GIRK_ ratio is defined as γ(Vm) = Δ*I*_10_/Δ*I*_500_ = *I*_GIRK_ (10 × 10^−9^ m)/*I*_GIRK_ (500 × 10^−9^ m). Right, statistics of γ(Vm) at the two holding potentials. γ(Vm) is potentiated at −100 mV versus −40 mV (***p < 0.001, Wilcoxon test, *n* = 62 cells). C) Similar to panel (B), but here in cells with D2R‐D131N mutation and GIRK1/4 co‐expression. The γ(Vm) shows no significant difference at Vm of −40 and −100 mV (*p*  = 0.19, n.s., not significant, Wilcoxon test, *n* = 43 cells). D) Log [QP] − normalized *I*_GIRK_ curves for D2R‐WT (left) and D131N (right) at holding potentials of −40 and −100 mV; the EC50 of QP for WT and D131N are calculated by fitting from log [agonist] versus normalized I_GIRK_ (normalized to *I*_GIRK_ evoked by 1000 × 10^−9^ m QP) responses. The dose‐dependent curve of the WT at −40 mV is significantly right shifted than at −100 mV (*n* = 17 cells) but not D131N (*n* = 26 cells), indicating that D2R‐WT is Vm‐dependent, while D131N mutation abolishes the initial Vm‐dependence. Inset, cartoon showing the D2R topology and positions of those transmembrane, charged and conserved amino acids in D2R, including D80, D114, and DRY motif‐D131and R132. D131 is identified as the voltage‐sensor in D2R by the reconstituted GIRK system via screening. Data are presented as the mean ± SEM (B, C). ***p < 0.001; *p* > 0.05, n.s., not significant.

Since D2R modulation of DA in vivo/ex vivo was AP frequency‐dependent and D2R modulation of quantal vesicular release in ACCs was also Vm‐dependent, we proposed that D2R per se is Vm‐sensitive, as we have recently reported the voltage‐dependence of another Gi‐coupled GPCR, P2Y_12_.^[^ ^14^ ^]^ To test this hypothesis, we introduced a Gi‐GPCR activation reporting system via GPCR‐Gi‐βγ‐GIRK current (*I*_GIRK_) recorded with whole‐cell patch‐clamp^[^ ^11^, ^14^, ^26^ ^]^ in which D2R and GIRK1/4 channels were co‐overexpressed in HEK293A cells (**Figure** **4**A). In the D2R‐Gi‐βγ‐*I*_GIRK_ bioassay, the D2R agonist QP triggered *I*_GIRK_ via the D2R‐Gi‐βγ‐*I*_GIRK_ signaling pathway and the *I*_GIRK_ showed QP dose‐dependence. A medium dose (10 × 10^−9^ m) and a saturation dose (500 × 10^−9^ m) of QP were used to induce *I*_GIRK_ to assess the voltage‐dependence of D2R (Figure 4A). The Vm of HEK293A cells was held at −40 or −100 mV, then *I*_GIRK_ was triggered by paired QP doses (10 × 10^−9^ m vs 500 × 10^−9^ m). The *I*_GIRK_ ratio was defined as γ(Vm) = Δ*I*_10_/Δ*I*_500_ = *I*_GIRK_ (10 × 10^−9^ m)/*I*_GIRK_ (500 × 10^−9^ m). γ(Vm) was larger at −100 mV than that at −40 mV in D2R‐expressing (D2R‐WT) cells (Figure 4B), suggesting the potentiation of *I*_GIRK_ via D2R activation. Thus, we concluded that Vm modulates D2R function and depolarization weakens D2R‐Gβγ signaling pathway. To confirm this, we introduced another complementary Gi‐GPCR activation reporting system, the D2R‐Gαi3q‐IP3‐[Ca^2+^]_i_ assay, to verify the D2R function, in which three plasmids expressing D2R, Gαi3q chimera, and Ca^2+^ indicator gCaMP3 were co‐transfected into the same HeLa cells. The Gαi3q chimera coupled with Gαi‐coupled receptors but signals transduced through the Gαq‐mediated PLC‐IP3‐[Ca^2+^]_i_ mobilization pathway.^[^ ^14^, ^27^ ^]^ This enabled us to efficiently visualize D2R‐Gαi activation‐induced [Ca^2+^]_i_ elevation with confocal Ca^2+^ imaging (Figure S13A,B, Supporting Information). Membrane depolarization with 70 × 10^−3^ m K^+^‐containing solution significantly inhibited the [Ca^2+^]_i_ elevation induced by the D2R agonist QP (Figure S13C–F and Movies S1–S3, Supporting Information), confirming that Vm modulates D2R function through the D2R‐Gi signaling pathway. Collectively, the two independent and complementary D2R downstream Gαi and Gβγ signal reporting systems validated that D2R itself is voltage‐dependent.

Next, we sought to identify the Vm‐sensing sites of D2R. Four amino‐acids—D80, D114, D131, and R132—were selected as candidate Vm‐sensing sites based on their charges, as well as transmembrane and conserved characteristics (Figure S14A,B, Supporting Information). When the negatively charged aspartic acid (D) or positively‐charged arginine (R) was mutated to the neutral asparagine (N), we found that the D2R‐D80N, D2R‐D114N, and D2R‐R132N mutants did not respond to its agonist QP, indicating that these D2R mutants were dysfunctional (Figure S14C, Supporting Information). Meanwhile, the D2R‐D131N mutant still responded to QP normally and mediated the *I*_GIRK_, while γ(Vm) was no longer larger at −100 mV than at −40 mV (Figure 4C), indicating that the D131N mutation abolished the Vm dependence of D2R. Moreover, the dose‐dependent curve of WT‐D2R at −40 mV was right‐shifted compared with that at −100 mV, and the phenomenon was missing when D131N mutant were used, suggesting that the Vm‐dependence of D2R is abolished by D131N mutation (Figure 4D). Besides, the D2R‐D131R mutation also abolished the Vm‐dependence (Figure S14D,E, Supporting Information), while the D2R‐D131E mutation (E, negatively charged glutamic acid) kept the Vm‐dependence but in the opposite direction (Figure S14F,G, Supporting Information) that of the WT (Figure 4B). Given that D2R has two isoforms,^[^ ^8^ ^]^ we next tested whether the voltage‐sensing site is shared by D2L with the reconstituted GIRK system. Strikingly, D2L showed similar Vm‐dependence like D2S (Figure 14H, Supporting Information), and this Vm‐dependence was abolished by all three tested mutations on D131 site (Figure 14I–K, Supporting Information), indicating the general voltage‐sensing mechanisms shared by both D2Rs. Taken together, D131 is the essential Vm‐sensitive site of D2R.

### 2.5 D2R Vm‐Sensor D131 Is Validated in Reconstituted ACCs

**Figure 5:**
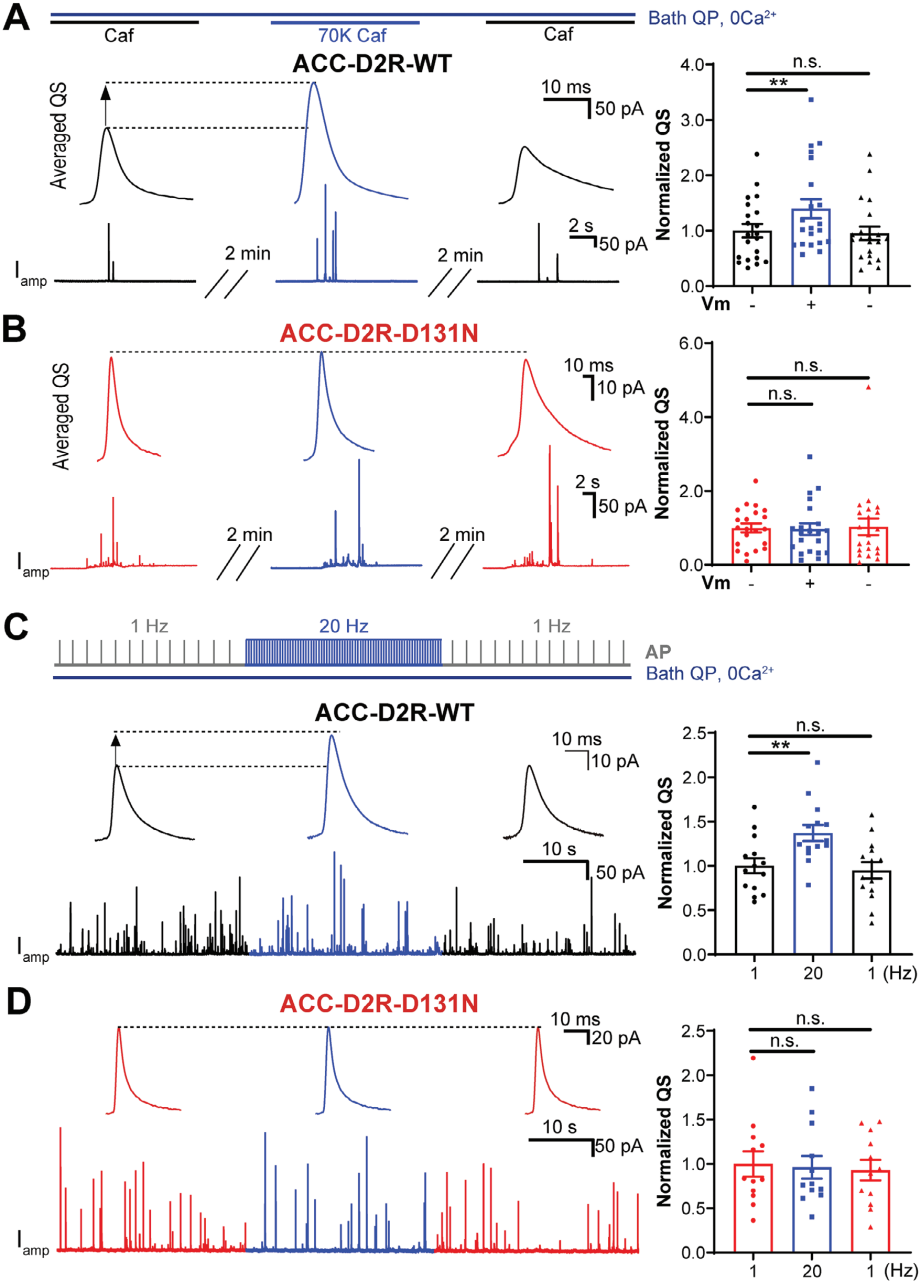
The D2R voltage‐sensing site (D131) validated by quantal vesicle release in reconstituted adrenal chromaffin cells (ACCs). A) Left, representative amperometric recordings (*I*_amp_) of quantal vesicle release triggered by 20 × 10^−3^ m caffeine (Caf) in ACCs overexpressing D2R‐WT (ACC‐D2R WT) bathed in 2 × 10^−6^ m QP and 0 Ca^2+^ solution. Insets show the averaged quantal size (QS) in each recording. Right, statistics of normalized QS. Results show that depolarization induced by 70 × 10^−3^ m KCl (70K) notably increases QS and the increment is abolished when 70K is removed (**p < 0.01, Friedman test, post hoc Dunn's multiple comparisons test, *n* = 21 cells). B) Similar to panel (A), but in RACCs overexpressing the D2R‐D131N mutation (ACC‐D2R‐D131N). Statistics show that the normalized QS does not change during 70K‐induced depolarization (*p*  = 0.25, n.s., not significant, Friedman test, post hoc Dunn's multiple comparisons test, *n* = 21 cells). C) Representative amperometric recording (left) and statistics (right) of quantal vesicle release triggered by whole‐cell dialysis of 1 × 10^−3^ m Ca^2+^ at 1 and 20 Hz AP frequency stimulation in ACC‐D2R‐WT cells bathed in QP. Insets show the averaged QS corresponding to the different AP frequencies. A high frequency (20 Hz) markedly increases QS compared to a low frequency (1 Hz) (**p < 0.01, one‐way ANOVA, post hoc Tukey's multiple comparisons test, *n* = 14 cells). D) Similar to panel (C), but in cells with the D2R mutation ACC‐D2R‐D131N. Normalized QS shows no difference between 1 and 20 Hz (*p*  = 0.96, n.s., not significant, one‐way ANOVA, post hoc Tukey's multiple comparisons test, *n* = 12 cells). Data are presented as the mean ± SEM (A–D). **p < 0.01; *p* > 0.05, n.s., not significant.

Since the D131N mutation abolished D2R Vm‐dependence in the D2R‐Gi‐βγ‐I_GIRK_ system, we next tested whether D131 served as the Vm‐sensor in the D2R‐ACC‐QS system. ACCs were bathed in 2 × 10^−6^ m QP‐containing and Ca^2+^‐free extracellular solution, quantal vesicular release was triggered by caffeine, and 70 × 10^−3^ m K^+^‐containing bath solution (70K) was used to depolarize the ACCs (**Figure** **5**A, protocol see also^[^ ^14^ ^]^). In blank control plasmid‐transfected ACCs, 70K‐induced depolarization showed no modulation on the quantal vesicular secretion (Figure S12E, Supporting Information). However, similar 70K‐depolarization significantly removed the QP inhibition on the QS of catecholamine release in D2R‐WT ACCs (Figure 5A), and this was abolished in D2R‐D131N ACCs (Figure 5B). Consistent with this, a high AP frequency (20 Hz) significantly reduced the QP inhibition on QS compared with 1 Hz stimulation in D2R‐WT ACCs (Figure 5C) but not in D2R‐D131N ACCs (Figure 5D). These findings suggest that the D131 site is essential for the Vm‐dependent D2R regulation of quantal vesicular release in ACCs.

### 2.6 Voltage‐D2R Effect Contributes up to Half of Total Voltage‐Induced DA Release In Vivo

**Figure 6:**
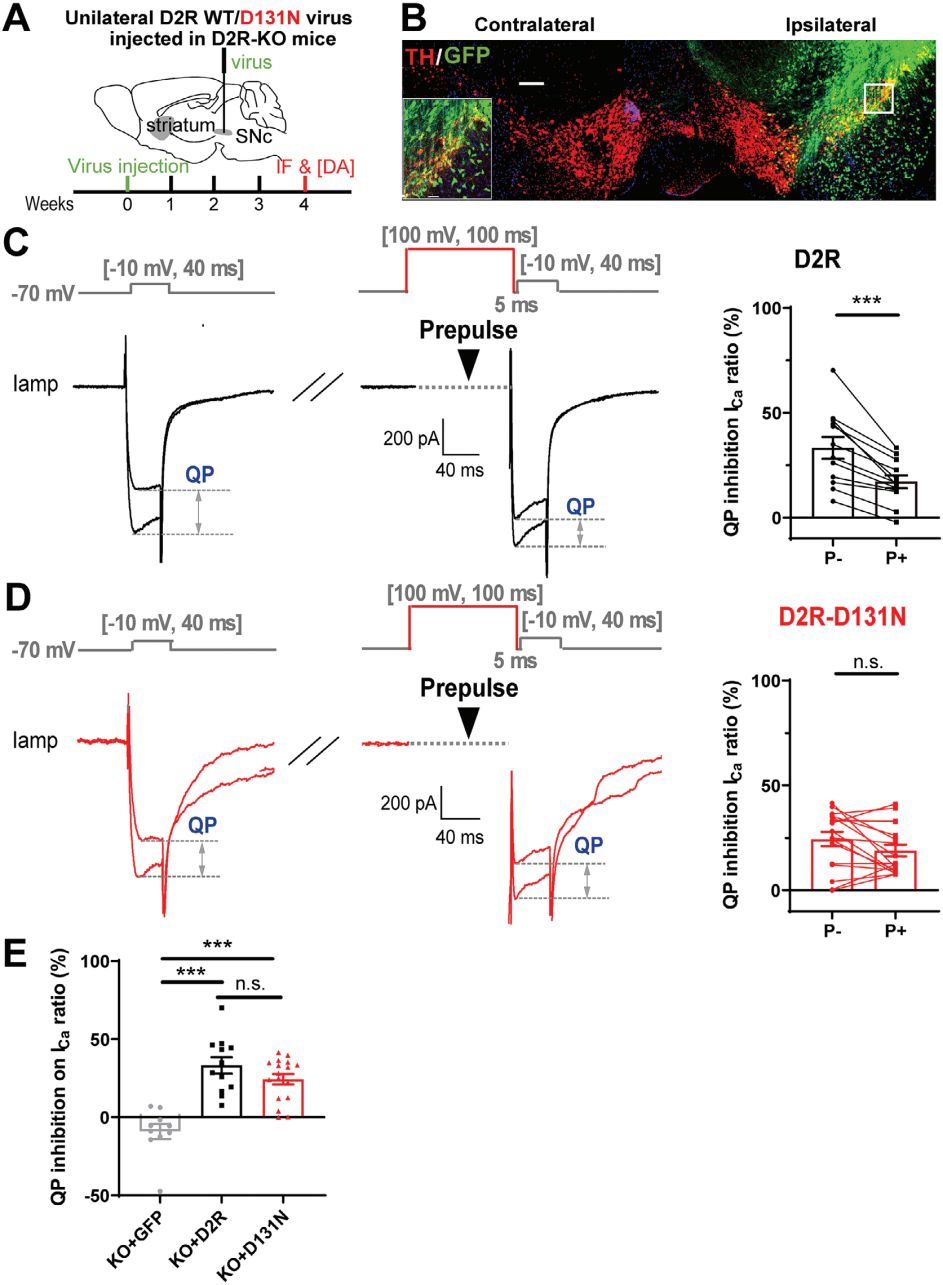
Validation of D2R‐D131 as voltage‐sensing site mediating the calcium channel facilitation in the striatum in situ. A) Diagram of unilateral injection of D2R‐WT and D131N AAV into the SNpc of D2R‐KO mice. Four weeks after virus injection, the anterograde AAV infects both the DA somata (SNpc) and terminal (striatum) areas. B) TH immunostaining (red) in contralateral/ipsilateral (without/with AAV) SNpc slice of a D2R‐KO mouse (scale bar, 200 µm). Inset, enlargement of demarcated SNpc region (scale bar, 50 µm). C,D) Representative traces and statistics showing the inhibitory effect of QP on *I*_Ca_ with or without prepulse depolarization in D2R‐KO mice overexpressing D2R (C) or D2R‐D131N (D). D2R rescued both the inhibitory effect of QP on *I*_Ca_ and the prepulse relieving of QP‐inhibition, while the neutralized D131N‐D2R mutant failed to rescue the alleviation effect (QP inhibition on *I*_Ca_ ratio, for D2R, P−: 33.26% ± 5.180% vs P+: 17.14% ± 3.033%; for D2R‐D131N, P−: 24.42% ± 3.332% vs P+: 19.00% ± 2.835%; paired Student's *t*‐test, *n* = 12 for D2R, *n* = 17 for D131N). E) Statistics showing that both D2R and mutant D131N similarly rescued the inhibitory effect of QP on *I*_Ca_ compared to D2R‐KO (QP inhibition on *I*_Ca_ ratio, GFP: −9.098% ± 4.795% vs D2R: 33.26% ± 5.180% vs D2R‐D131N: 24.42% ± 3.332%; one‐way ANOVA test, *n* = 12 for GFP, *n* = 12 for D2R, *n* = 17 for D131N). Data are presented as the mean ± SEM. ***p < 0.001; *p* > 0.05, n.s., not significant.

Since D2R‐D131 has been confirmed to be the Vm‐sensor in both the D2R‐Gi ‐βγ‐GIRK and D2R‐ACC‐QS reconstituted systems, we next tested whether D131 still works in the physiological DA system in vivo. We injected D2R‐WT, D2R‐D131N, or control GFP‐expressing adeno‐associated virus (AAV) into the substantia nigra pars compacta (SNpc) of D2R‐KO mice to evaluate their rescue effects on DA release (**Figure** **6**A). Four weeks after virus injection, immunofluorescence showed the efficient infection of DA neurons as shown by the GFP co‐localization in tyrosine hydroxylase (TH, red)‐positive cells (Figure 6B). Consistent with the in vitro assay, D2R‐WT rescued both the inhibitory effect of QP on *I*_Ca_ and the prepulse relieving of QP‐inhibition, while the neutralized D2R‐D131N mutant failed to rescue the alleviation effect (Figure 6C–E), validating the voltage‐dependent modulation of VGCCs by D2R. Then, we tested the D131 Vm‐sensing function for D2R‐regulated DA release in striatal slices with CFE amperometric recordings. We found that the QP regulation of DA release was weakened by high AP frequency in D2R‐WT‐rescued D2R‐KO mice (Figure S15A–C, Supporting Information), while AP frequency failed to modulate QP effect when D2R‐D131N was used (Figure S15D–F, Supporting Information). Thus, AP frequency‐dependent modulation of DA release was removed when the D131 site was neutralized (Figure S15, Supporting Information).

**Figure 7:**
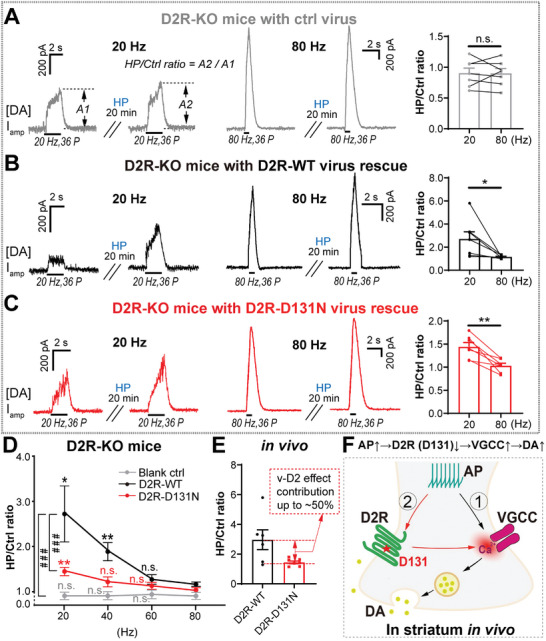
Validation of D2R‐D131 as voltage‐sensing site mediating activity‐dependent modulation of DA release in the striatum in vivo. A) Representative amperometric recordings and statistics of evoked [DA] in vivo before and after HP treatment at 20 and 80 Hz E‐stim in D2R‐KO mice overexpressing control virus tagged with GFP (Blank ctrl). B) Similar to panel (A), but in D2R‐KO mice overexpressing D2R‐WT virus (D2R‐WT‐rescue). C) Similar to panel (B), but in D2R‐KO mice overexpressing D2R‐D131N virus (D2R‐D131N‐rescue). D) AP frequency‐dependence of [DA] amplitude ratio (HP/Ctrl) in WT‐rescue and D131N‐rescue mice (for comparison among groups^#^, two‐way ANOVA test; for comparison in groups^*^, paired Student's *t*‐test; *n* = 7 mice for each group). E) Statistics of in vivo [DA] amplitude ratio (HP/Ctrl) at 20 Hz in D2R‐KO mice overexpressing D2R‐WT and D2R‐D131N virus [paired Student's *t*‐test for (C)–(E), G; *n* = 7 mice for each group]. Notably, the HP/Ctrl ratio decreased almost half in D2R‐D131N rescue compared to D2R‐WT, indicating that the Vm‐D2R effect contributes up to ≈50% of voltage induced DA release at the physiological 20 Hz frequency in vivo. F) Model of voltage‐sensitive D2R regulation of DA release in the striatal dopaminergic terminal in vivo. Collectively, voltage (AP) can increase DA release by disinhibiting D2R via Vm↑ → D2R (D131) ↓→ VGCCs↑ → DA release↑, a novel pathway (②) coexisting with the canonical Ca^2+^‐triggered vesicular secretion pathway (①) for DA release following physiological APs in the striatum in vivo. Data are presented as the mean ± SEM (C–G). *p < 0.05; **p < 0.01; ***^/###^ *p* < 0.001; *p* > 0.05, n.s., not significant.

For the final validation of D2R‐D131 in the physiological DA system, we next applied in vivo DA recording. First, in D2R‐KO mice injected with the blank control virus, as expected, the HP/Ctrl ratios at different AP frequencies ranging from 20 to 80 Hz were all the same (HP/Ctrl ≈ 1), confirming the lacking of frequency‐dependent modulation of [DA] in the absence of D2R (**Figure** **7**A,D). Second, we found that HP differentially affected the [DA] evoked by 20 Hz versus 80 Hz in D2R‐WT virus‐rescued D2R‐KO mice and the HP/Ctrl ratio was significantly larger at 20 Hz than at 80 Hz (Figure 7B). Furthermore, the HP/Ctrl ratio showed a significant frequency‐dependent reduction at different AP frequencies from 20 to 80 Hz in D2R‐WT rescued D2R‐KO mice (Figure 7D, black line). However, in D2R‐D131N‐rescued D2R‐KO mice, the HP/Ctrl ratio at 20 Hz was still higher than that at 80 Hz, but with a much‐reduced difference compared with the WT‐rescue group (Figure 7C,D). Consistent with this, the HP/Ctrl ratio also showed an AP frequency‐dependent reduction in D2R‐D131N‐rescued mice (Figure 7D, red line), but the tendency was greatly weakened compared with that in D2R‐WT‐rescued mice (Figure 7D, black line). The HP/Ctrl ratio of DA release in vivo decreased 47% at 20 Hz in D2R‐D131N rescued mice compared to D2R‐WT (Figure 7E). Together, these findings suggest that D2R‐D131 is the Vm‐sensor for AP frequency‐dependent modulation of DA overflow, and the voltage effect on D2R (Vm‐D2R effect) contributes up to 50% of total voltage‐induced DA release in vivo (Figure 7E). Collectively, we report a pivotal physiological function of voltage dependent‐D2R in mammalian central nervous system in vivo: APs↑ → D2R (D131) ↓ → VGCCs↑ → DA release↑ (Figure 7F). This GPCR (D2R) dominant noncanonical (AP→D2R→VGCC) pathway (contributes up to 50% of DA release) coexists with the canonical (AP→VGCC) Ca^2+^‐triggered vesicular secretion pathway for DA release following physiological APs in the striatum in vivo, which enriches and updates the canonical theory of DA release triggered by VGCCs.

## 3 Discussion

DA release induced by Vm depolarization or APs is fundamental in the DA system, and it relies on Ca^2+^ influx through VGCCs. Here, we report a novel mechanism in which Vm/APs not only triggers DA release, but also modulates DA release by directly targeting Vm‐sensitive D2Rs (Gi‐coupled GPCRs) via the Vm‐sensing site D131. This was validated by using three independent reconstituted D2R activation systems, as well as real‐time electrochemical recordings in striatal slices and in vivo.

The major finding of the present work is that different AP firing patterns (low versus high frequencies) of DA neurons differentially regulate the autoreceptor‐D2R modulation of DA release in the striatum in vivo. It has been well‐established that AP triggers neurotransmitter release via the Ca^2+^ influx through VGCCs, and thus the Ca^2+^‐dependent vesicular exocytosis. Recent studies also revealed that AP triggers Ca^2+^‐independent vesicular exocytosis through the voltage‐mediated conformational changes of VGCC‐SNARE fusion machinery in primary sensory neurons, sympathetic nerve system, and synaptic transmission in central nerve system.^[^ ^28^ ^]^ However, whether and how the firing pattern alteration modulates neurotransmission remain largely unknown. Our study provides in vivo evidence of Vm regulation of DA release through D2R by expressing WT and mutant D2R in the SNpc of D2R‐KO mice. By monitoring DA release in the striatum, we reinforce the conclusion that AP or membrane potential also modulates DA release by deactivating presynaptic D2R autoreceptor. It is worth noting that the HP/Ctrl ratio decreased almost half in D2R‐D131N compared to D2R‐WT rescued mice (Figure 7), indicating that the Vm‐D2R effect contributes up to 50% of APs‐triggered DA release in vivo. Nonetheless, the residual AP frequency‐dependent modulation of DA release in the voltage‐insensitive D2R‐D131N mutant (Figure 7) suggests that other factors may also be involved in the AP‐modulation of D2Rs. For example, independent of the voltage‐sensing site of D2R per se, the dynamics of extracellular DA concentration may also be altered by AP‐frequency and thus contribute to the modulation of presynaptic D2R activation.^[^ ^29^ ^]^

Regarding the molecular mechanism underlying the Vm‐dependent D2R modulation of DA release, three in vitro reconstituted D2R activation reporting systems were introduced by overexpressing exogenous D2R in native ACCs (D2R‐ACC‐QS assay), nonexcitable HEK 293A cells (D2R‐Gi‐βγ‐GIRK assay), and nonexcitable HeLa cells (D2R‐Gαi3q‐IP3‐[Ca^2+^]_i_ assay). These assays provide direct evidence for the Vm‐dependence of D2R and the Vm‐sensing site of D2R. As we have reported previously, the quantal size of vesicle release is regulated by the endogenous Gi‐GPCRs (P2Y_12_ and somatostatin receptors) via the Gi‐βγ signaling pathway in ACCs,^[^ ^24^ ^]^ here we constructed an exogenous Gi‐GPCR activation reporting system in native ACCs by overexpressing D2R, making the D2R‐ACC‐QS assay an ideal readout/tool for studying the function of D2R. Further high‐precision D2R‐Gi‐βγ‐GIRK and high‐throughput D2R‐Gαi3q‐IP3‐[Ca^2+^]_i_ bioassays were based on two complementary GPCR downstream Gβγ and Gα pathways. D2R function was shown to be Vm‐dependent in these reconstituted systems, which together strongly indicate that D2R itself is Vm‐dependent. In principle, these D2R activation reporting assays can be used to study the Vm dependence and/or other functions of D2R, and can also be generalized to the functional study of other GPCRs.

The voltage sensitivity of GPCRs has been reported previously.^[^ ^30^ ^]^ Especially, Sahlholm and colleagues have tested the voltage‐dependency of D2R in the modulation of GIRK current in *Xenopus laevis* oocytes in vitro, and they have identified that some serine residues (particularly S193) in TM5 were involved in the voltage sensitive potency for DA binding due to their contact to hydroxyl groups.^[^ ^30^, ^31^ ^]^ However, whether the properties can be reproduced under physiological conditions (at the levels of single cell, slice in situ, and in vivo) and whether it is functionally involved in synaptic transmission remain unknown. The present work, for the first time, demonstrated the voltage‐dependent modulation of D2R on DA release in mouse brain striatum in situ and in vivo, not only defined critical roles of D2R in the modulation of vesicular exocytosis but also identified a new voltage‐sensing site (D131) of D2R in mammalian cells. The negatively‐charged aspartate residue of TM3 (D131) was defined to be the Vm‐sensing site of D2R by using the D2R‐Gi‐βγ‐GIRK assay, confirmed by the D2R‐ACCs‐QS assay, and validated in the striatum ex vivo and in vivo by using rescue approaches in D2R‐KO mice. Taken together, D131 is the Vm‐sensing site of D2R responsible for the AP frequency‐dependent DA release in situ and in vivo. Considering that TM3‐DRY‐D131 in D2R is involved in the highly‐conserved and charged DRY motif in class A GPCRs,^[^ ^32^ ^]^ and recently we also reported another Vm‐sensitive GPCR/P2Y_12_ and its Vm‐sensing site TM3‐DRY‐D127,^[^ ^14^ ^]^ we propose the voltage‐dependence as a general property of class A GPCR.

The D2 receptor gene encodes two splicing variants, D2S and D2L. D2S is predominantly expressed in presynaptic DA neurons, functioning as an auto‐receptor to inhibit neural excitation and DA release, whereas D2L primarily mediates DA transmission in post‐synapse and thus contributes to the circuit‐specific modulation of various physiological processes.^[^ ^8^ ^]^ We strikingly found that the voltage‐dependency is shared by both presynaptic D2S and postsynaptic D2L, suggesting that the firing pattern alteration of postsynaptic D2R‐neurons per se may further contribute to DA physiological processes by integrating other circuit inputs onto DA transmission.^[^ ^33^ ^]^ Furthermore, the role of voltage‐sensitive D2R also contributes to understanding the function of other class A GPCRs and provides new insights into the physiological and pathological changes of GPCR signaling pathways.

Consistent with the well‐established D2R regulation on VGCCs, D2R agonist QP showed inhibition on *I*_Ca_, which was absent in D2R‐KO DA neurons. Interestingly, a prepulse depolarization removed the blockade effect of QP on *I*_Ca_, which was abolished by D2R‐KO. Furthermore, application of the LTCCs blocker nifedipine also completely abolished the AP frequency‐dependent modulation of DA release by D2R agonist QP. Importantly, D2R overexpression (OE) rescued both the inhibitory effect of QP on I_Ca_ and the prepulse relieving of QP‐inhibition in D2R‐KO DA neurons, while the neutralized D131N‐D2R mutant failed to rescue the alleviation effect, validating the voltage‐dependent modulation of VGCCs by D2R. Thus, we propose that the voltage‐dependent modulation of D2R on DA release is mediated by VGCCs via the pathway APs↑→D2R (D131) ↓→VGCCs↑→DA release↑. Collectively, these findings confirmed the inhibitory effect of D2R on VGCC activation and further demonstrated that higher frequency of APs lead to the disinhibition of D2R on VGCCs and thus mediate VGCC facilitation, which is critical for the activity‐dependent modulation of DA release by D2R. In addition to its well‐known “first role” to gate VGCCs, membrane depolarization or high‐frequency APs also directly disinhibit D2R autoreceptor activity, leading to the increase of DA release in the striatum in vivo. In addition to functioning as a chemical‐sensing GPCR, D2R (D131) also acts as a novel voltage sensor to instantly enhance striatal DA release in vivo through the Vm‐disinhibition of D2R inhibitory function, which enriches and updates the canonical pathway of Vm‐VGCCs to modulate striatal DA release.

Since that the Vm‐dependence of D2R plays a pivotal role in regulating striatal DA release in vivo, whether and how this phenomenon contributes to the vast DA‐dependent motor and nonmotor behaviors in both health (such as reward, modes, and cognition) and diseased brain (such as depression, drug addiction, Parkinson's disease, and schizophrenia) deserve systematic investigation in future.^[^ ^1^, ^3^ ^]^ The universality of the voltage‐dependence of GPCRs may also shed new light in the application of transcranial magnetic stimulation (TMS) and low intensity transcranial electrical stimulation (tES) in the clinical therapies of various of mental health disorders (such as anxiety, depression, and drug addiction).^[^ ^34^ ^]^

## 4 Experimental Section

### Animals and Materials

The D2R‐KO mice on a C57BL/6 background^[^ ^35^ ^]^ were gifts from Prof. Jiawei Zhou (Institute of Neuroscience, CAS, China) and kept in the Animal Center of Peking University under a 12‐h light/dark cycle. The C57BL/6J mice were purchased from Beijing Vital River Laboratory Animal Technology Co., Ltd. All experimental procedures were approved by the Peking University Animal Use and Care Committee and performed according to the guidelines of the Association for Assessment and Accreditation of Laboratory Animals guidelines (IACUC approval no. IMM‐ZhouZ‐13). Mice in 2–6 months old were used for all in vivo and slice electrochemistry experiments. The collagenase (C9891), hyaluronidase (H3506), poly‐l‐lysine (P1399), haloperidol (H1512‐5G), quinpirole (MFCD01321054), nomifensine (N1530), raclopride (R121), and caffeine (93784) were purchased from Sigma Aldrich. The Neon transfection reagent (MPK1096) and Lipo2000 (11668027) were from Invitrogen. The Vigofect (T001) was from Vigorous Biotechnology and DHβE (Cat#2349) was from Tocris. Rabbit anti‐D2R (Cat#AB5084P) and Rabbit anti‐TH (Cat#AB152) were from Millipore. Goat anti‐rabbit IgG Alexa Fluor 488 (Cat#A‐11034) and Goat anti‐rabbit IgG Alexa Fluor 594 (Cat#A‐11037) were from Thermo Fisher Scientific.

### Plasmids

D2R and related mutation plasmids were generated from an original human D2R prokaryotic expression vector which was a gift from Yulong Li (Peking University, China). The D2R site mutations D131N, D131E, D131R, D80N, D114N, and R132N were produced by PCR using a QuikChange II Site‐Directed Mutagenesis Kit (Agilent Technologies). A bicistronic plasmid expressing GIRK1 and GIRK4 subunits (GIRK1/4 channels) was provided by Diomedes Logothetis (Virginia Commonwealth University). For in vivo rescue, AAV carrying D2R, D2R‐D131N, or control (pHBAAV‐hsyn‐T2A‐ZsGreen) were produced by Hanbio Biotechnology Co., Ltd. (Shanghai, China). All constructs were verified by DNA sequencing.

### In Vivo Electrochemistry

Amperometric DA recording in vivo was performed as previously described.^[^ ^36^ ^]^ The anesthetized mouse (urethane 1.5 g kg^−1^, i.p.) was fixed on a stereotaxic instrument (SR‐6N, Narishige, Japan). Body temperature was maintained at 37 °C using a heating blanket (KEL‐2000, Nanjing Kell, China). [DA] was recorded in the dorsal striatum in vivo using an electrochemical CFE. The recording CFE 400 µm in length was holding at 780 mV to oxidize the substance and implanted in the caudate–putamen of the dorsal striatum (CPu: 1.1 mm AP; 1.7 mm ML, 3.4 mm DV). A bipolar stimulating electrode (Plastics One Inc., USA) was implanted in the MFB (2.1 mm AP, 1.1 mm ML, 4.0–5.0 mm DV). An Ag/AgCl reference electrode was placed in the contralateral cortex. Electrical stimuli were generated by a high‐voltage isolator (SYS‐A365D, WPI, USA) as a train of several biphasic square‐wave pulses (0.6 mA, 1 ms duration); a total of 36 pulses at 20/40/60/80/100 Hz were delivered at 5‐min intervals for each stimulation. The amperometric signal was amplified by a patch‐clamp amplifier (PC2C, INBIO, Wuhan, China), low pass‐filtered at 50 Hz (F1000, INBIO, Wuhan, China), and recorded by a PClamp software (INBIO, Wuhan, China). Haloperidol (0.4 mg kg^−1^) or quinpirole (8 mg kg^−1^) were administered via i.p. injection.

### Striatal Slice Electrochemistry

An amperometry DA recording assay was performed in slices as previously described.^[^ ^19a^ ^]^ The anesthetized mouse (urethane 1.5 g/kg, i.p.) was transcardially perfused with ice‐cold artificial cerebrospinal fluid (ACSF) cutting solution containing (in mm): 110 C_5_H_14_NClO, 2.5 KCl, 0.5 CaCl_2_, 7 MgCl_2_, 1.3 NaH_2_PO_4_, 25 NaCO_3_, 25 glucose. Then the brain was rapidly removed and cut into 300 µm coronal slices on a vibratome (Leica VT 1000s; Nussloch, Germany). The striatal slices were collected in recording ACSF containing (in mm): 125 NaCl, 2.5 KCl, 2 CaCl_2_, 1.3 MgCl_2_, 1.3 NaH_2_PO_4_, 25 NaCO_3_, 10 glucose. The slices were incubated at 37 °C for recovery then kept at room temperature for recording. A bipolar stimulating electrode was used to trigger DA release while a 200‐µm long CFE held at 780 mV was used for recording in the dorsolateral caudate region of the striatum. Electrical stimuli were generated by a Grass S88 K stimulator (Astro‐Med, USA) as single or a train of several biphasic square‐wave pulses (0.6 mA, 0.2 ms duration); a total of 6 pulses at 2/100 Hz was delivered at 3‐min intervals for each stimulation. Amperometric signals were recorded using an EPC9/2 amplifier and Pulse software (HEKA Electronic, Lambrecht/ Pfalz, Germany). Quinpirole (0.5 × 10^−6^ m) was applied by a local puffing system.

### Cell Culture and Transfection

Rat adrenal chromaffin cells (RACCs) were prepared as described previously.^[^ ^24b^ ^]^ Adrenal glands were isolated from anesthetized rats (10% chloral hydrate, 0.8–1 mL/150 g), cut into 10 pieces after removing the cortex. The pieces were incubated with collagenase (1 mg mL^−1^) and hyaluronidase (2 mg mL^−1^) for 40 min at 37 °C then triturated gently through a 200‐µL pipette tip. RACCs were isolated after centrifugation, then transfected with D2R (2 µg) and GFP (0.5 µg) plasmids using a Neon electroporation system (Invitrogen, MPK1096) according to the manufacturer's instructions. RACCs were plated on coverslips pre‐coated with 0.1% poly‐l‐lysine and cultured at 37 °C under 5% CO_2_ for 24 h before experiments. HEK293A cells were cultured in DMEM with 10% FBS at 37 °C under 5% CO_2_ and transfected with D2R (1 µg), a bicistronic plasmid expressing GIRK1 and GIRK4 subunits (1 µg), and GFP (0.5 µg) plasmids (per 3.5 cm diameter dish) by VigoFect (Vigorous Biotechnology Beijing Co.) After 24 h of expression, cells were placed on coverslips and the GIRK current was recorded.

### Single‐Cell Electrochemistry

Single‐vesicle quantal release of catecholamines from RACCs was recorded by a highly sensitive CFE as previously described.^[^ ^24^, ^37^ ^]^ A patch pipette filled with an intracellular solution containing 1 × 10^−3^ m Ca^2+^ was used to trigger catecholamine release. The Vm was held at −70/0 mV or 1/20 Hz APs by whole‐cell patch clamp and amperometric signals were recorded using an EPC10/2 amplifier and PatchMaster software (HEKA Electronic, Lambrecht/ Pfalz, Germany). Cells were kept in normal extracellular solution containing (in mm): 145 NaCl, 2 CaCl_2_, 2.8 KCl, 1 MgCl_2_, 10 HEPES (pH 7.4) with 2 × 10^−6^ m quinpirole in the bath.

### Electrophysiology

An EPC10/2 amplifier with PatchMaster software was used for whole‐cell patch clamp recordings as previously described.^[^ ^38^ ^]^ A patch pipette was filled with an intracellular solution containing (in mm): 100 K^+^‐aspartate, 40 KCl, 5 NaCl, 7 MgCl_2_, 10 EGTA, 0.025 GTP, 5 Na^+^‐ATP, 20 HEPES (pH 7.2). Cells were kept in normal extracellular solution during experiments. For GIRK current recording, a high K^+^ buffer was used as the extracellular solution containing (in mm): 2.4 NaCl, 2 CaCl_2_, 140 KCl, 1 MgCl_2_, 10 HEPES (pH 7.4), then GIRK currents were measured by patch clamp at a holding potential of −40/−100 mV;^[^ ^11^ ^]^ 10 or 500 × 10^−9^ m quinpirole was used to activate the D2R‐mediated GIRK current.

### In Vivo AAV Infection

Stereotactic viral injections were performed as previously described.^[^ ^39^ ^]^ Adult (≈25 g) D2R‐KO mice were anesthetized with avertin (20 mL kg^−1^, i.p.) and fixed in a stereotaxic instrument. Craniotomies were performed to cause minimal damage to cortical tissue and then leveled by bregma and lambda landmarks. Body temperature was maintained at 37 °C using a heating blanket. A total of 0.5 µL viral suspension (titer: 10^12^ V g mL^−1^) was unilaterally injected into the SNc (3 mm posterior to bregma, 1.25 mm lateral, 4 mm ventral to the dura). Afterward, the scalp was sutured and mice were placed on a 37 °C plate for recovery then kept for 30–45 d of viral expression before in vivo DA recordings.

### Immunohistochemistry

Immunohistochemistry was performed as previously described.^[^ ^19a^ ^]^ Mice were anesthetized with 25% urethane then perfused with 0.9% saline followed by 4% paraformaldehyde (PFA) The brain was removed and post‐fixed overnight in 4% PFA then dehydrated in 10%, 20%, and 30% sucrose at 4 °C. A series of coronal sections (40 µm thick) across the midbrain and striatum were cut on a Leica cryostat. The sections were permeabilized with 0.3% Triton X‐100 in PBS for 5 min at room temperature. After blocking with 2% bovine serum albumin (BSA) in PBS, the samples were incubated with the primary antibody rabbit anti‐TH (AB152, Merck Millipore) at 4 °C overnight. After washing with 2% BSA, samples were incubated for 1 h with the secondary antibody (Alexa Fluor 594 goat anti‐rabbit IgG, A11037, Invitrogen) at room temperature. Nuclei were visualized by DAPI staining then the sections were mounted on coverslips immersed in 50% glycerol. For RACCs, cells were prefixed in 4% paraformaldehyde for 15 min, washed three times with PBS, then permeabilized with 0.3% Triton in PBS for 3 min. After the cells were incubated with 2% BSA for 1 h, they were incubated with the primary antibody rabbit anti‐D2R (AB5084P, Millipore) overnight at 4 °C. Then they were washed with 2% BSA in PBS, and incubated with the secondary antibody (Alexa Fluor 488 goat anti‐rabbit IgG, A11034, Invitrogen). After that, the cells were mounted on coverslips immersed in 50% glycerol. Fluorescence images were acquired on an inverted confocal microscope (Zeiss 710, Germany) and analyzed with ImageJ (National Institutes of Health, Bethesda, MD).

### Statistical Analysis

All experiments and protocols were performed with side‐by‐side controls and in random order, and were replicated at least three times. Data were analyzed with IGOR Pro software (WaveMetrics, USA). Data are presented as the mean ± SEM. and *n* represents the number of independent experiments. All tests were conducted using Prism V7.0 (GraphPad Software, Inc.) and SPSS 20.0 (Statistical Package for the Social Sciences). If the data passed the normality test, then statistical comparisons were performed with Student's *t*‐test or one‐way ANOVA followed by Tukey's multiple comparisons test. If the data did not pass the normality test, then the Wilcoxon matched‐pairs signed rank test (Wilcoxon test) or Friedman test followed by Dunn's multiple comparisons test was used for data analysis. Statistical tests were two‐tailed and *p* < 0.05 was set as statistically significant (*p < 0.05, **p < 0.01, ***p < 0.001).

## Conflict of Interest

The authors declare no conflict of interest.

## Author Contributions

X.X.S., L.L.Y., and Z.J.Q. contributed equally to this work. Z.Z., Q.F.Z., C.H.W., B.L., P.L.Z., and X.X.S. designed the research; X.X.S., L.L.Y., and Z.J.Q. performed experiments and analyzed data; M.Y., G.Q.C., X.W., J.L., X.J.K., H.D.X., L.Z., Y.L.L., M.G., X.Y.D., Y.Q.H., Z.H.L., L.Y.S., Q.L.W., R.Y.J., L.W., M.Q.H., Y.W., R.H., Y.M.L., Q.H.W., S.J.S., S.G., Q.L., H.F.S., L.H.Z., S.R.W., and. F.P.Z. performed experiments; Q.F.Z., Z.Z., and C.H.W. wrote the paper.

## Supporting information



Supporting Information

Supplemental Movie 1

Supplemental Movie 2

Supplemental Movie 3

## Data Availability

The data that support the findings of this study are available on request from the corresponding author. The data are not publicly available due to privacy or ethical restrictions.
