# Supplementary material for: Action Potential Firing Patterns Regulate Dopamine Release via Voltage‐Sensitive Dopamine D2 Autoreceptors in Mouse Striatum In Vivo
Source: Adv Sci (Weinh). 2024 Dec 27;12(7):2412229. doi: 10.1002/advs.202412229 (PMC11831442; doi:10.1002/advs.202412229)
Supplement: Supplementary file 1 — Supporting Information [file ADVS-12-2412229-s003.docx]

Supporting Information for

**Action potential firing patterns regulate dopamine release via voltage-sensitive dopamine D2 autoreceptors in mouse striatum *in vivo***

*Xiaoxuan Sun^*^, Lili Yin, Zhongjun Qiao, Muhammad Younus, Guoqing Chen, Xi Wu, Jie Li, Xinjiang Kang, Huadong Xu, Li Zhou, Yinglin Li, Min Gao, Xingyu Du, Yuqi Hang, Zhaohan Lin, Liyuan Sun, Qinglong Wang, Ruiying Jiao, Lun Wang, Meiqin Hu, Yuan Wang, Rong Huang, Yiman Li, Qihui Wu, Shujiang Shang, Shu Guo, Qian Lei, Haifeng Shu, Lianghong Zheng, Shirong Wang, Feipeng Zhu, Panli Zuo^*^, Bing Liu^*^, Changhe Wang^*^, Quanfeng Zhang^*^, Zhuan Zhou^*^*

X. Sun, L. Yin, Z. Qiao, M. Younus, G. Chen, X. Wu, J. Li, X. Kang, H. Xu, L. Zhou, Y. Li, M. Gao, X. Du, Y. Hang, Z. Lin, L. Sun, Q. Wang, R. Jiao, L. Wang, M. Hu, Y. Wang, R. Huang, Y. Li, Q. Wu, S. Shang, S. Guo, Q. Lei, H. Shu, L. Zheng, S. Wang, F. Zhu, P. Zuo, B. Liu, C. Wang, Q. Zhang, Z. Zhou

State Key Laboratory of Membrane Biology, National Biomedical Imaging Center and Institute of Molecular Medicine, College of Future Technology; Peking-Tsinghua Center for Life Sciences; and PKU-IDG/McGovern Institute for Brain Research, Peking University, Beijing 100871, China

E-mail: [zzhou@pku.edu.cn](mailto:zzhou@pku.edu.cn); [zhangquanfeng@pku.edu.cn](mailto:zhangquanfeng@pku.edu.cn); [changhecool@163.com](mailto:changhecool@163.com); [lbing0926@163.com](mailto:lbing0926@163.com); [panlizuo@gmail.com](mailto:panlizuo@gmail.com); [sunxiaoxuan0729@vip.163.com](mailto:sunxiaoxuan0729@vip.163.com))

X. Sun

Peking University Sixth Hospital, Peking University Institute of Mental Health, NHC Key Laboratory of Mental Health (Peking University), National Clinical Research Center for Mental Disorders (Peking University Sixth Hospital), Beijing 100191, China

C. Wang

Neuroscience Research Center, Institute of Mitochondrial Biology and Medicine, Key Laboratory of Biomedical Information Engineering of Ministry of Education, School of Life Science and Technology, Xi’an Jiaotong University, Xi’an 710049, China

C. Wang

Department of Neurology, the Second Affiliated Hospital of Xi’an Jiaotong University, Xi’an 710004, China.

C. Wang

Key Laboratory of Medical Electrophysiology, Ministry of Education of China, Collaborative Innovation Center for Prevention and Treatment of Cardiovascular Disease, and the Institute of Cardiovascular Research, Southwest Medical University, Luzhou 646000, China.

*Corresponding authors

**This PDF file includes:**

Figure S1-S15

Movie S1-S3

**Other Supporting Information for this manuscript include the following:**

Movie S1-S3


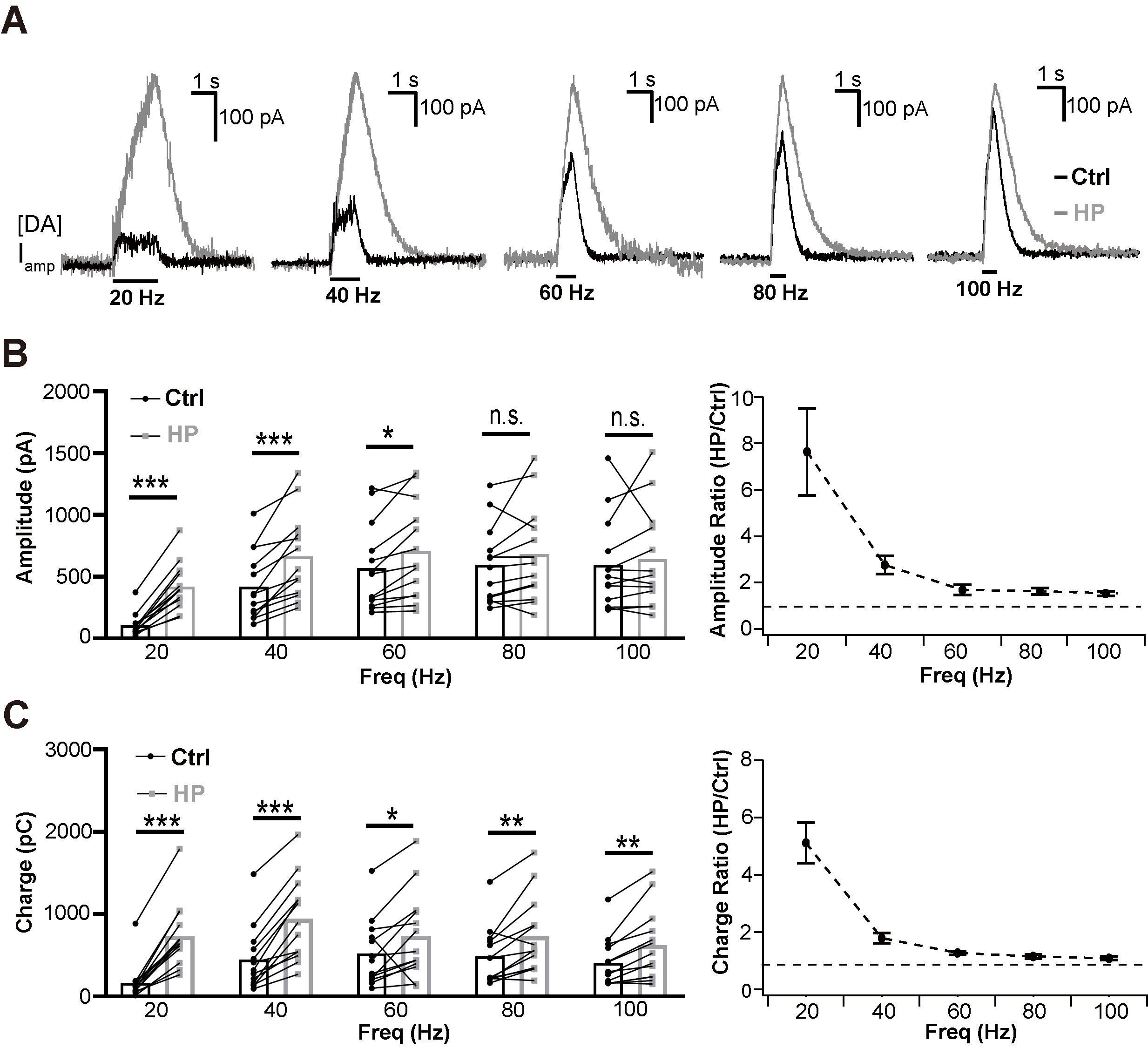


**Figure S1 (related to Figure 1). The effect of the D2R antagonist haloperidol on [DA] is frequency-dependent**

A) Representative traces of evoked [DA] [DA] before (black) and after (gray) treatment with the D2R antagonist haloperidol (HP, 0.4 mg/kg, *i.p*.) under a series of AP frequencies of stimulation (from 20 Hz to 100 Hz) in C57 mice. B, C) Left, the increasing effect of HP on [DA] amplitude (B) and charge (C) is more evident at low AP frequency, while high AP frequency removes the modulation of [DA] signals by HP. Right, AP frequency-dependence curve of [DA] amplitude (B) and charge (C) (paired Student’s *t*-test, *n* = 13 mice for B and C). Data are presented as the mean ± SEM (B, C). **p* <0.05; ***p* <0.01; ****p* < 0.001; *p* > 0.05, n.s., not significant.


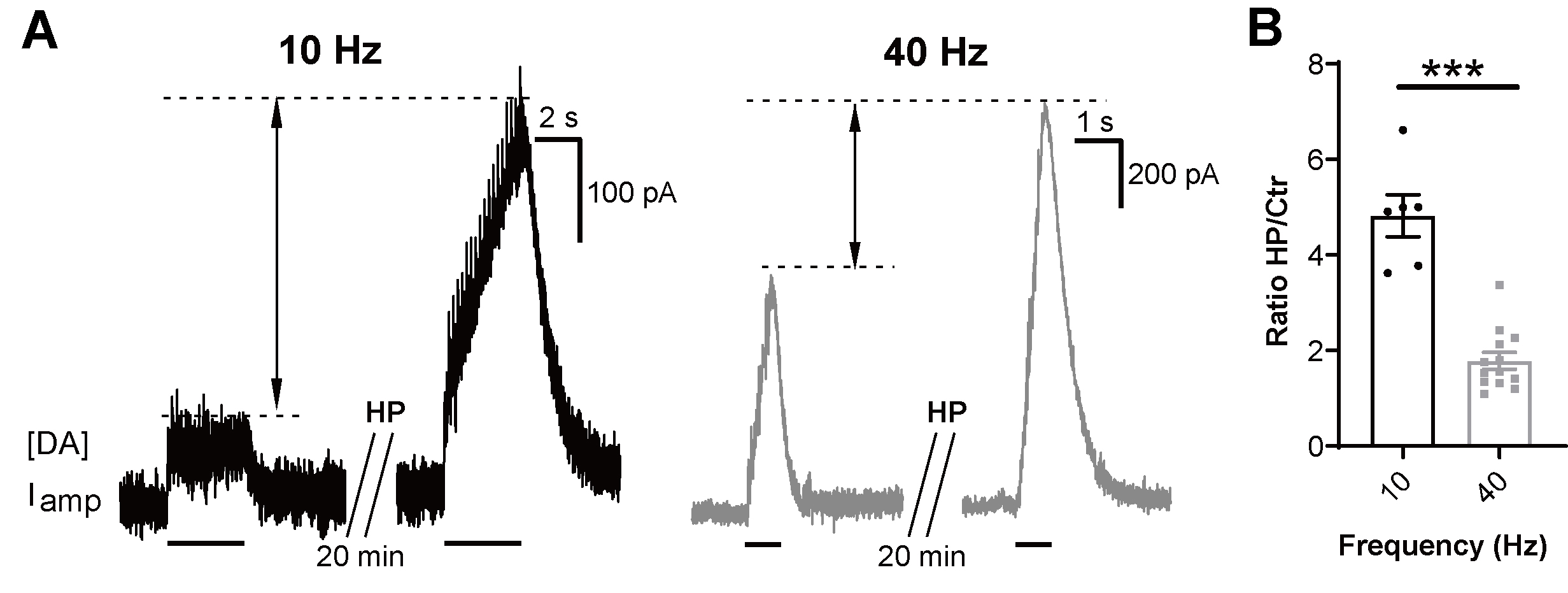


**Figure S2 (related to Figure 1). The voltage-sensitive modulation of D2R on [DA] occurs under physiological conditions**

A, B) Representative amperometric recordings and statistics of evoked [DA] in the striatum *in vivo* before and after HP (0.4 mg/kg, *i.p.*) treatment under 10 Hz or 40 Hz electrical stimulation. The ratio of HP effect on [DA] amplitude at 40 Hz Estim decreased greatly compared to that of 10 Hz (unpaired Student’s *t*-test, *n* = 7 for 10 Hz and *n* = 13 for 40 Hz). Data are presented as the mean ± SEM (B), ****p* < 0.001.


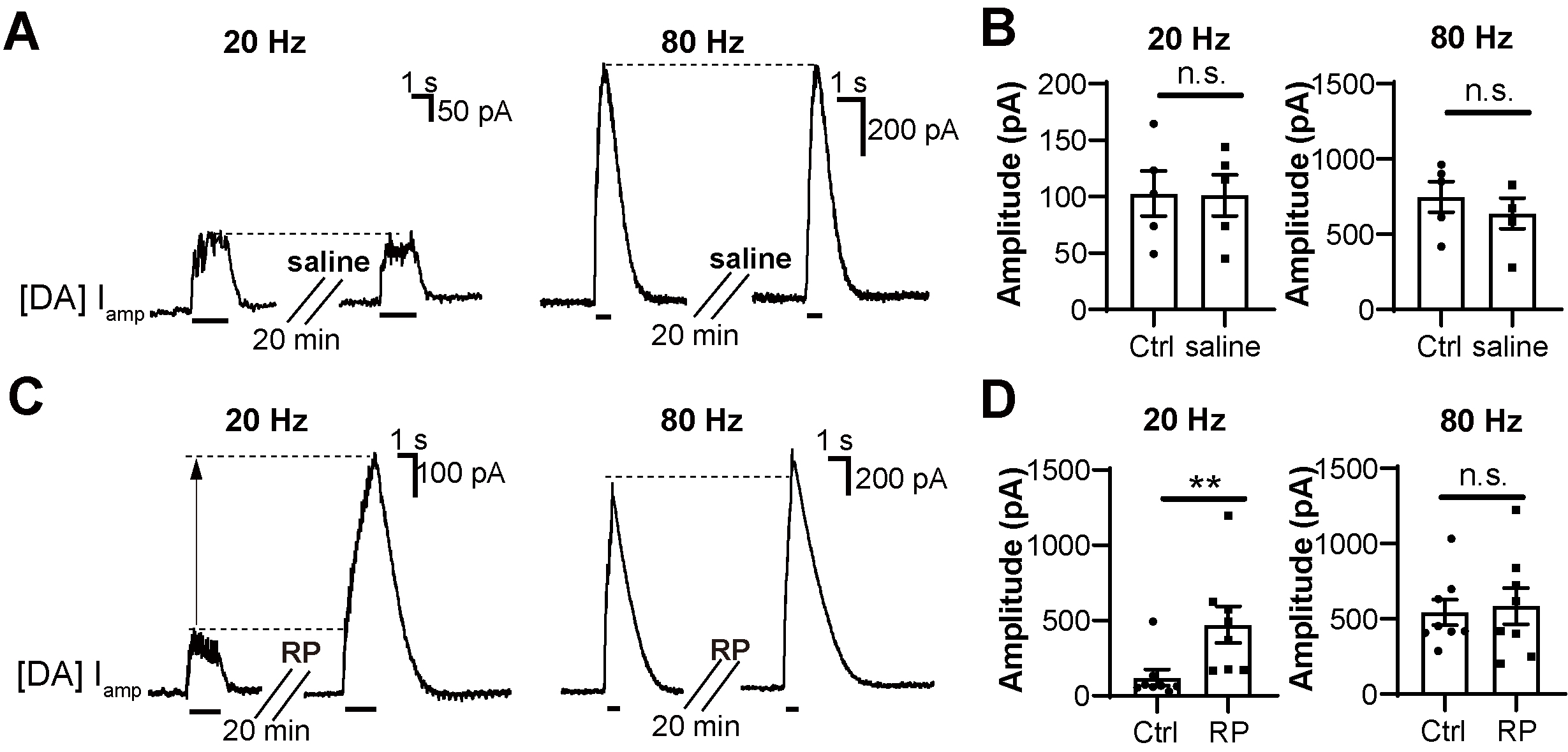


**Figure S3 (related to Figure 1). The effect of D2R antagonist raclopride (RP) on [DA] is frequency-dependent**

A, B) Representative amperometric recordings and statistics of evoked [DA] before and after control saline treatment under 20 Hz and 80 Hz electrical stimulation in C57 mice. Saline shows no modulating effect on [DA] amplitude at both 20 Hz and 80 Hz (paired Student’s *t*-test, *p* = 0.78 for 20 Hz, *p* = 0.09 for 80 Hz, *n* = 5 mice). C, D) Representative amperometric recordings and statistics of evoked [DA] before and after raclopride (RP, 2 mg/kg, *i.p.*) treatment under 20 Hz or 80 Hz electrical stimulation in C57 mice. Statistics show that RP significantly increases [DA] amplitude at 20 Hz, while it does not alter [DA] amplitude at 80 Hz (Wilcoxon test, ***p* < 0.01 for 20 Hz; paired Student’s *t*-test, *p* = 0.47 for 80 Hz; *n* = 8 mice). Data are presented as the mean ± SEM (B and D). ***p*< 0.01; *p* > 0.05, n.s., not significant.


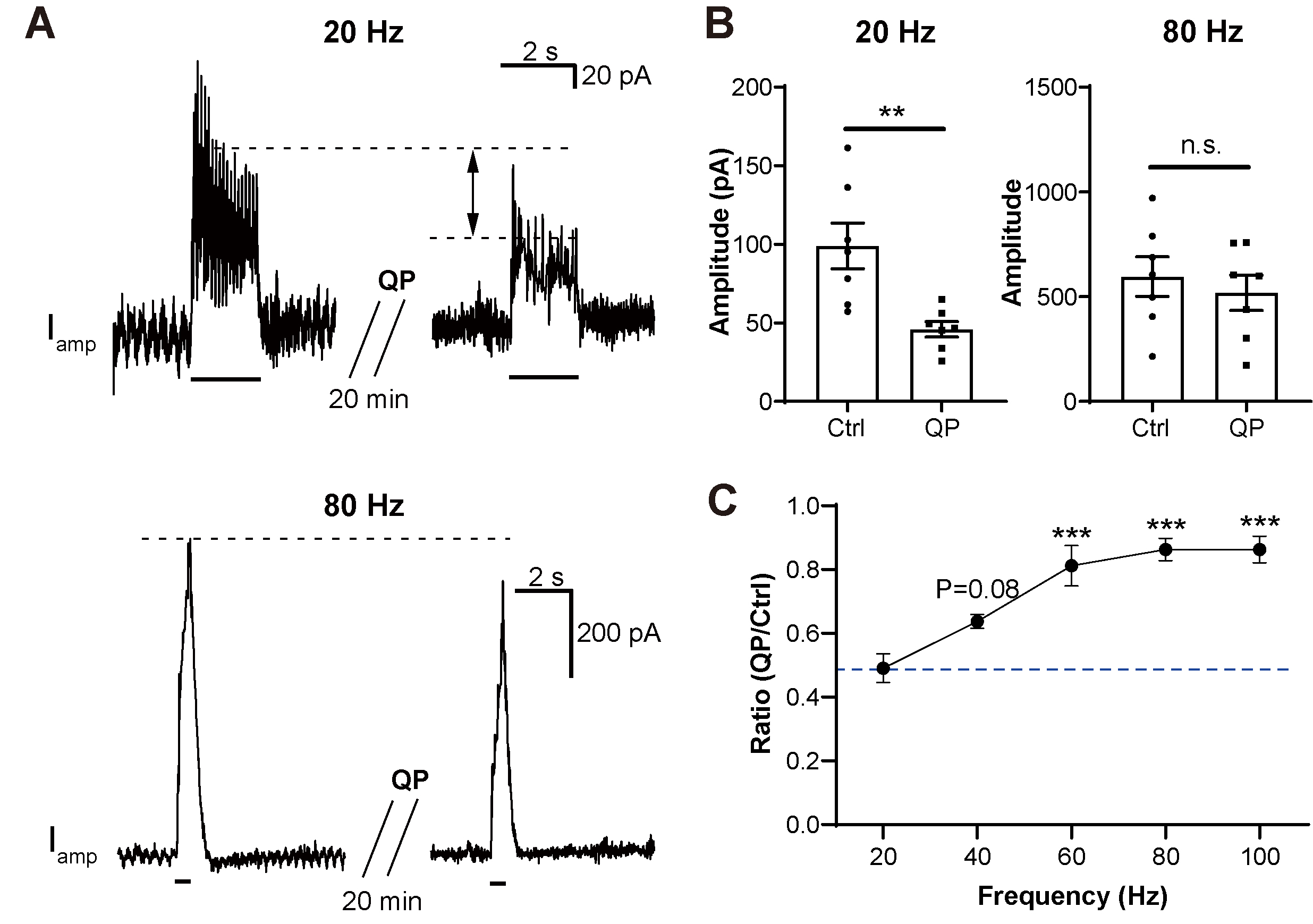


**Figure S4 (related to Figure 1). The effect of D2R agonist quinpirole (QP) on [DA] is frequency-dependent**

A, B) Representative amperometric recordings and statistics of evoked [DA] before and after quinpirole (QP, 8 mg/kg, *i.p.*) treatment under 20 Hz or 80 Hz electrical stimulation in C57 mice. Statistics show that QP significantly inhibited DA release at 20 Hz, while it failed to alter [DA] at 80 Hz. (Unpaired Student’s *t*-test, ***p* < 0.01 for 20 Hz; *p* = 0.5554 for 80 Hz; *n* = 7 mice). C) The inhibition curve of QP on DA release is AP frequency-dependent, the inhibitory effect of QP is more evident at lower AP frequency. (One-way ANOVA followed by Tukey’s pairwise comparisons with 20-Hz stimulus, *n* = 7 mice). Data are presented as the mean ± SEM. ***p* < 0.01; ****p* < 0.001.


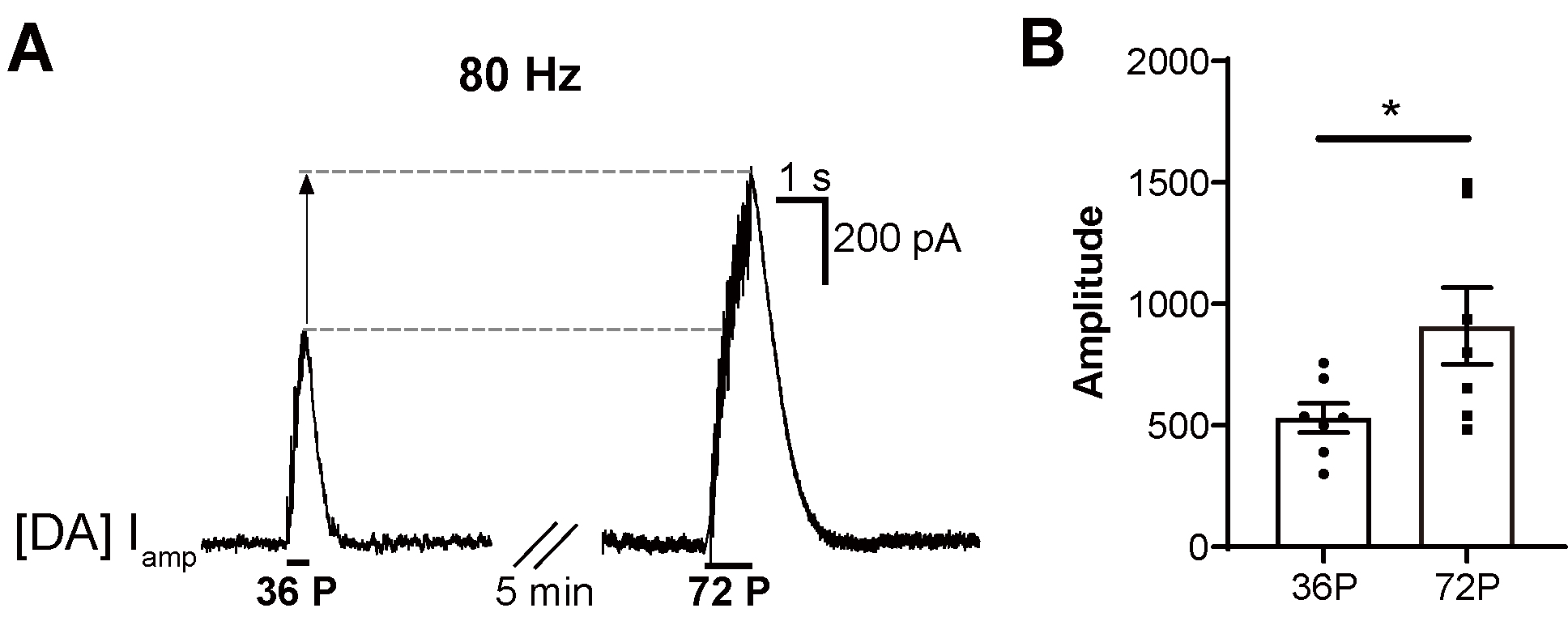


**Figure S5 (related to Figure 1). [80 Hz, 36 pulses] stimulation-evoked [DA] is not saturated**

A) Representative amperometric recordings of [DA] evoked by [80 Hz, 36 pulses] and [80 Hz, 72 pulses] electrical stimulation in C57 mice. B) Statistics of [DA] evoked by 72 pulses is much higher than that by 36 pulses (paired Student’s *t*-test, *n* = 7 mice). Data are presented as the mean ± SEM. **p* < 0.05.


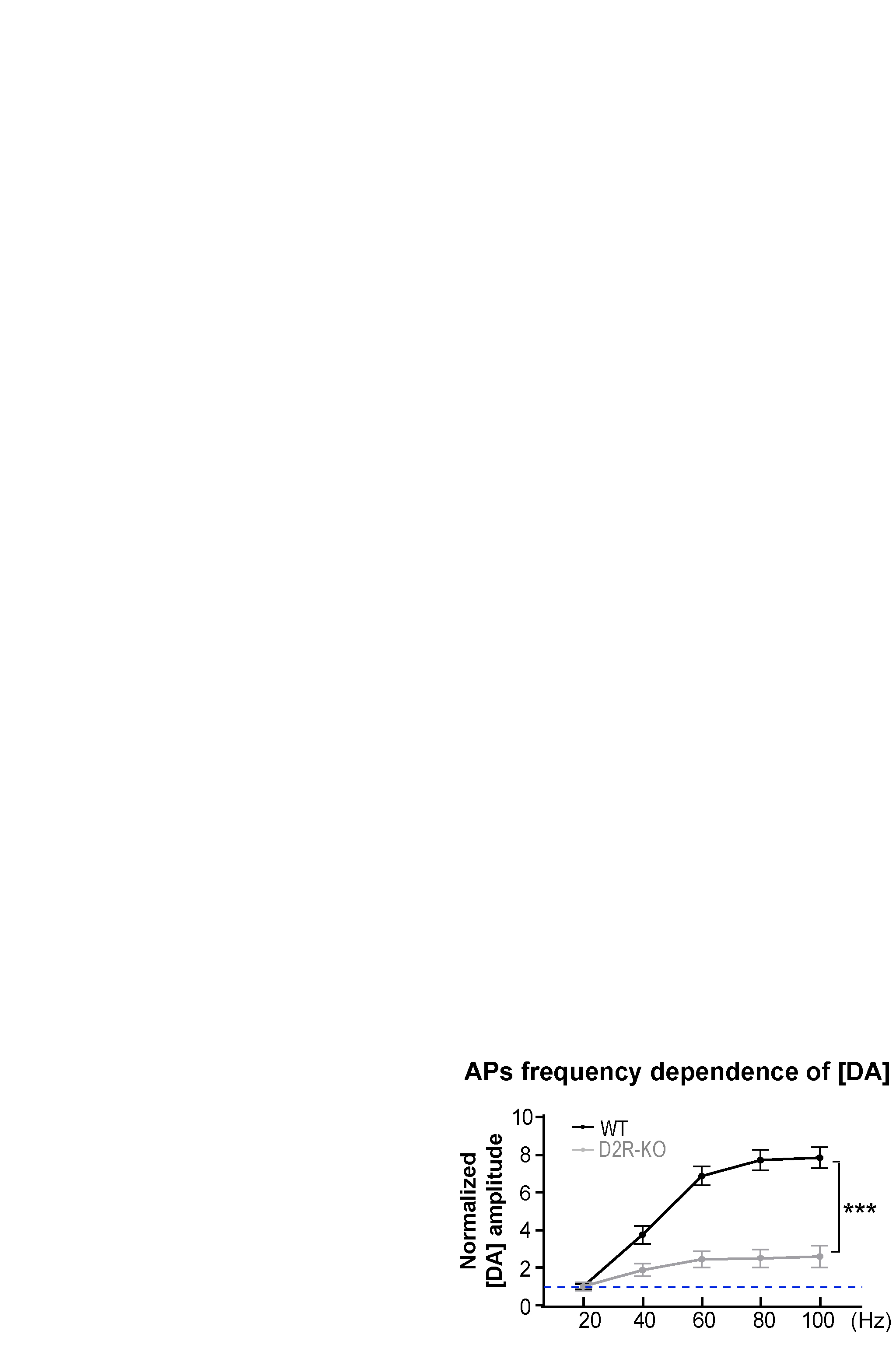


**Figure S6 (related to Figure 1). AP frequency-dependence of [DA] in WT and D2R-KO mice**

AP frequency-dependence of [DA] (normalized to [DA] evoked by 20 Hz) in WT and D2R-KO mice. Along with the increment of AP frequency from 20 Hz to 100 Hz, the evoked [DA] is gradually increased in both WT and D2R-KO mice, but the slope is much steeper in WT compared with D2R-KO mice (Two-way RM ANOVA, *n* = 9 pairs for each group). Data are presented as the mean ± SEM. ***p* < 0.01, ****p* < 0.001; *p* > 0.05, n.s., not significant.


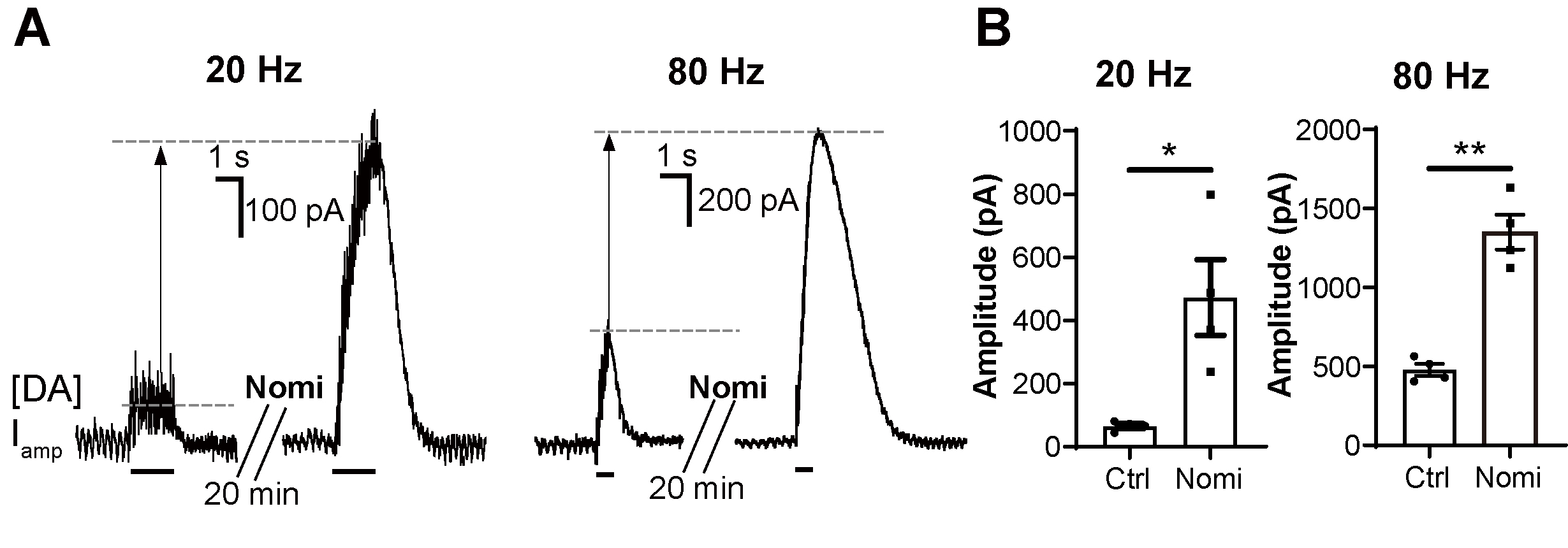


**Figure S7 (related to Figure 1). The regulatory effect of the DAT blocker nomifensine (Nomi) on [DA] shows no frequency dependence**

A, B) Representative amperometric recordings and statistics of evoked [DA] before and after nomifensine (Nomi, 8 mg/kg, *i.p.*) treatment under 20 Hz or 80 Hz electrical stimulation in C57 mice. Nomi increases [DA] amplitude at both 20 Hz and 80 Hz (paired Student’s *t*-test, **p* < 0.05 for 20 Hz; ***p* < 0.01 for 80 Hz, *n* = 4 mice). Data are presented as the mean ± SEM. **p* < 0.05, ***p*< 0.01.


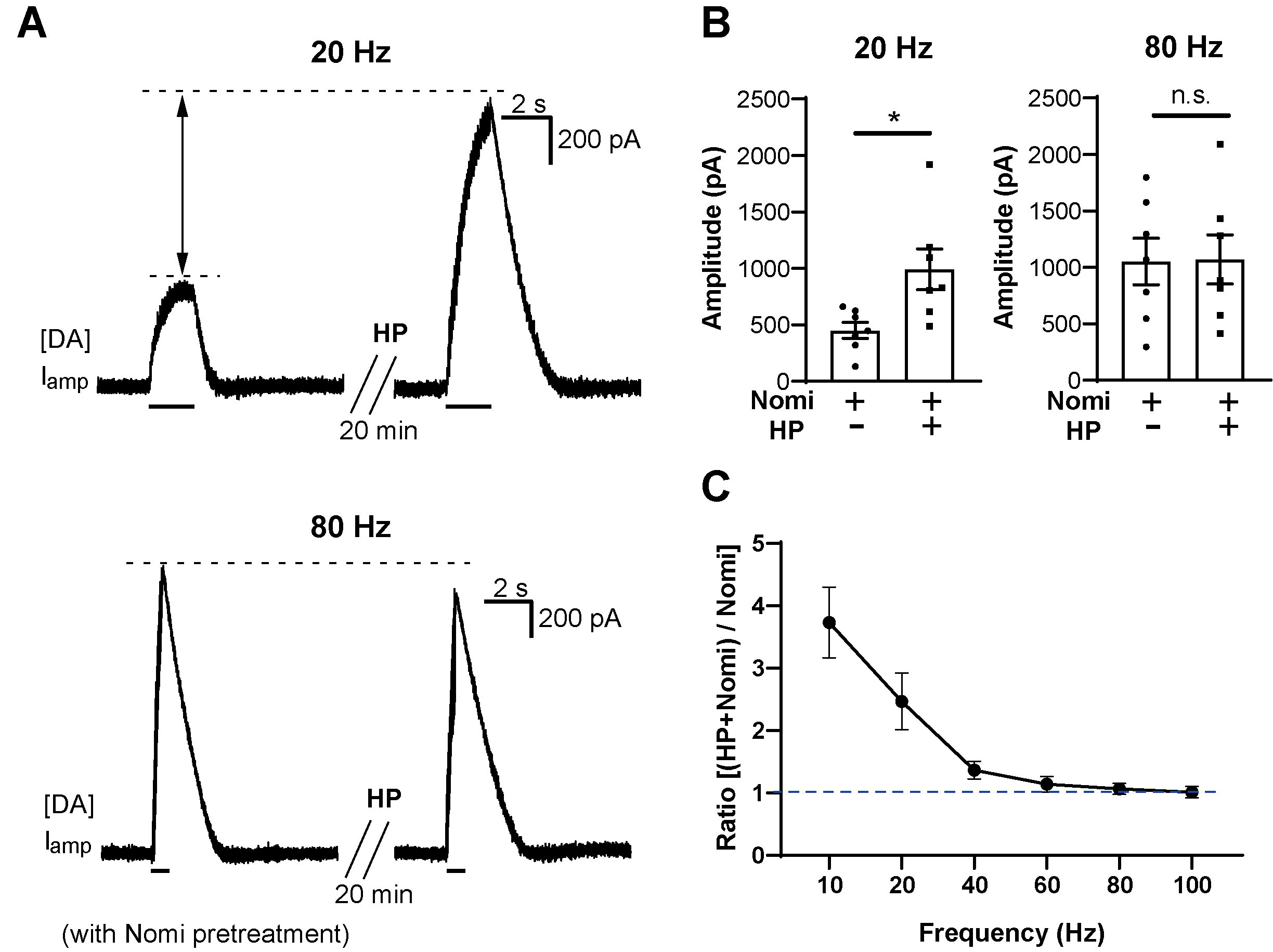


**Figure S8 (related to Figure 1). The facilitatory effect of haloperidol on [DA] remains frequency-dependent in the presence of DAT blocker nomifensine**

A, B) Representative amperometric recordings and statistics of evoked [DA] before and after HP (0.4 mg/kg, *i.p.*) treatment under 20 Hz or 80 Hz electrical stimulation with Nomi pretreatment (8 mg/kg, *i.p.*) in C57 mice. (Unpaired Student’s *t*-test, **p* < 0.05 for 20 Hz; *p* = 0.9525 for 80 Hz; *n* = 7 mice). C) Statistics showing the AP frequency-dependence of [DA] with the increment of AP frequency from 10 Hz to 100 Hz in the presence of nomifensine (One-way ANOVA followed by Tukey’s pairwise comparisons with 10 Hz stimulus). Data are presented as the mean ± SEM. ****p* < 0.001; **p* < 0.05.


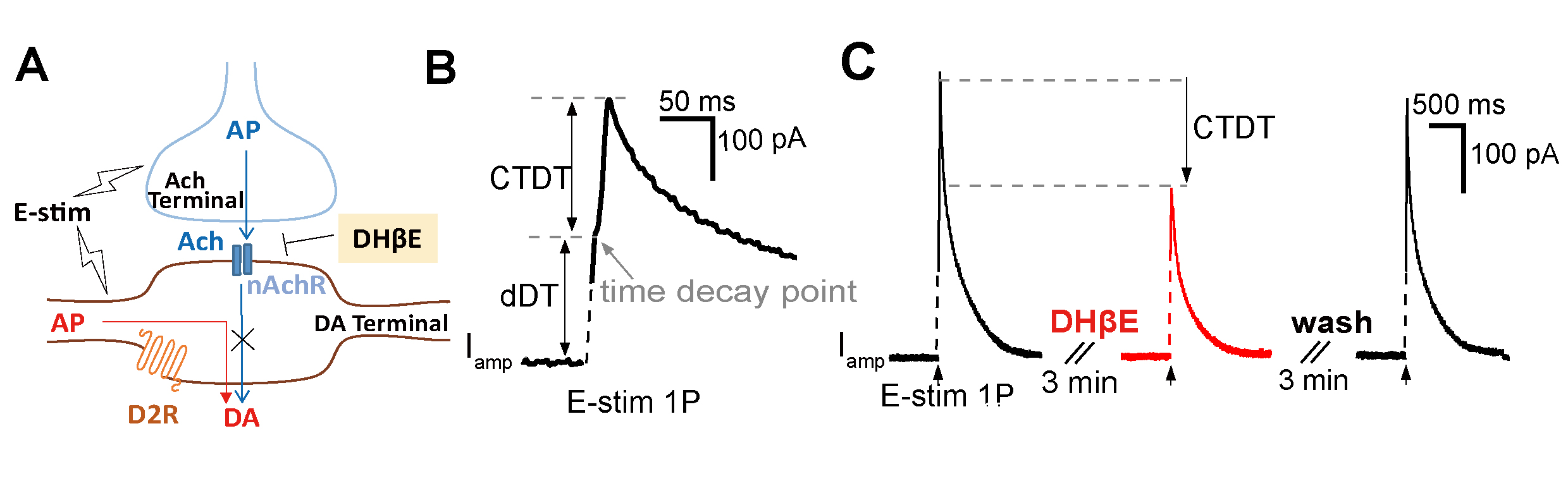


**Figure S9 (related to Figure 2). DHβE inhibits DA release from the cholinergic transmission pathway in** **striatal slices**

A) Diagram of two distinct DA release pathways from presynaptic terminals in striatal slices. Following a single electrical field stimulation (1 pulse), both direct DA transmission (dDT, red line) and cholinergic transmission-induced DA transmission (CTDT, blue line) are activated. After application of dihydro-β-erythroidine hydrobromide (DHβE), a nicotinic acetylcholine receptor (nAChR) antagonist, the CTDT pathway is totally blocked. B) DA release from both dDT and CTDT pathways is evoked by 1 pulse of electrical stimulation (E-stim 1P) and are recorded by CFE in striatal slices. The two phases of DA release can be temporally distinguished, as dDT has shorter latency than CTDT. C) Representative traces of evoked DA release with 1 μM DHβE treatment under E-Stim 1P in C57 mice. The CTDT pathway is totally blocked by DHβE.


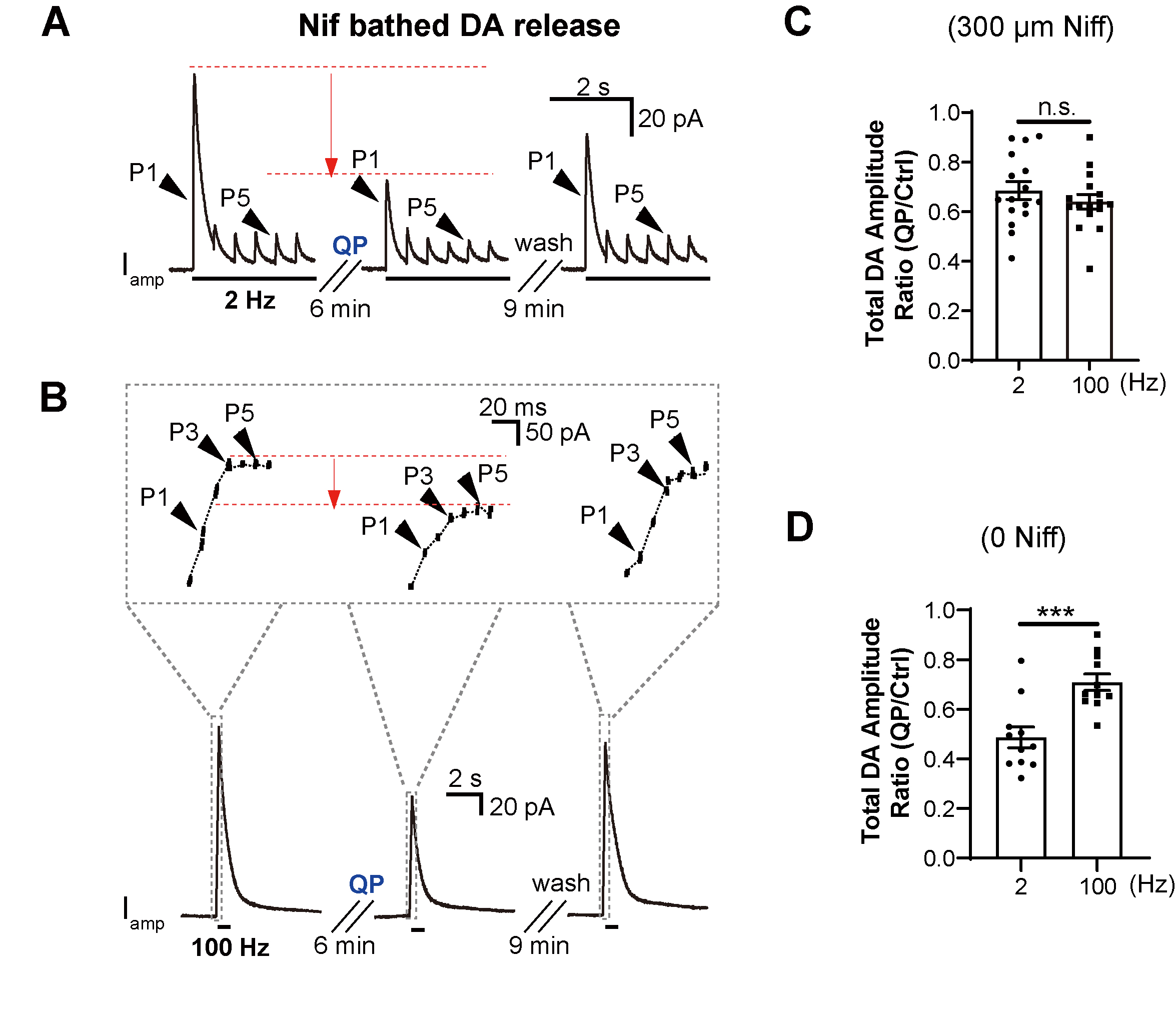


**Figure S10 (related to Figure 2). L-Type calcium channels (LTCCs) blocker nifedipine abolishes the AP-frequency modulation of D2R on DA release**

A, B) Evoked DA release (with E-stim patterns of 2 Hz 6 pulses *vs* 100 Hz 6 pulses) from striatal slices bathed in 300 μM nifedipine (Nif, a potent blocker of LTCCs) before and after QP treatment. The enlarged inset shows the detailed DA signals following 6 pulses (P1 to P6) of 100-Hz Estim. C, D) Statistics of QP effect on total DA release (summation of amplitude I_P1_+I_P2_+…+ I_P6_) at two E-stim frequencies in the presence (C) and absence (D) of Nif (paired Student’s *t*-test, *p* = 0.2727, n.s., *n* = 16 for C and *p* < 0.001, ***, *n* = 11 for D). Data are presented as the mean ± SEM. *p* >0.05, n.s., not significant.

**
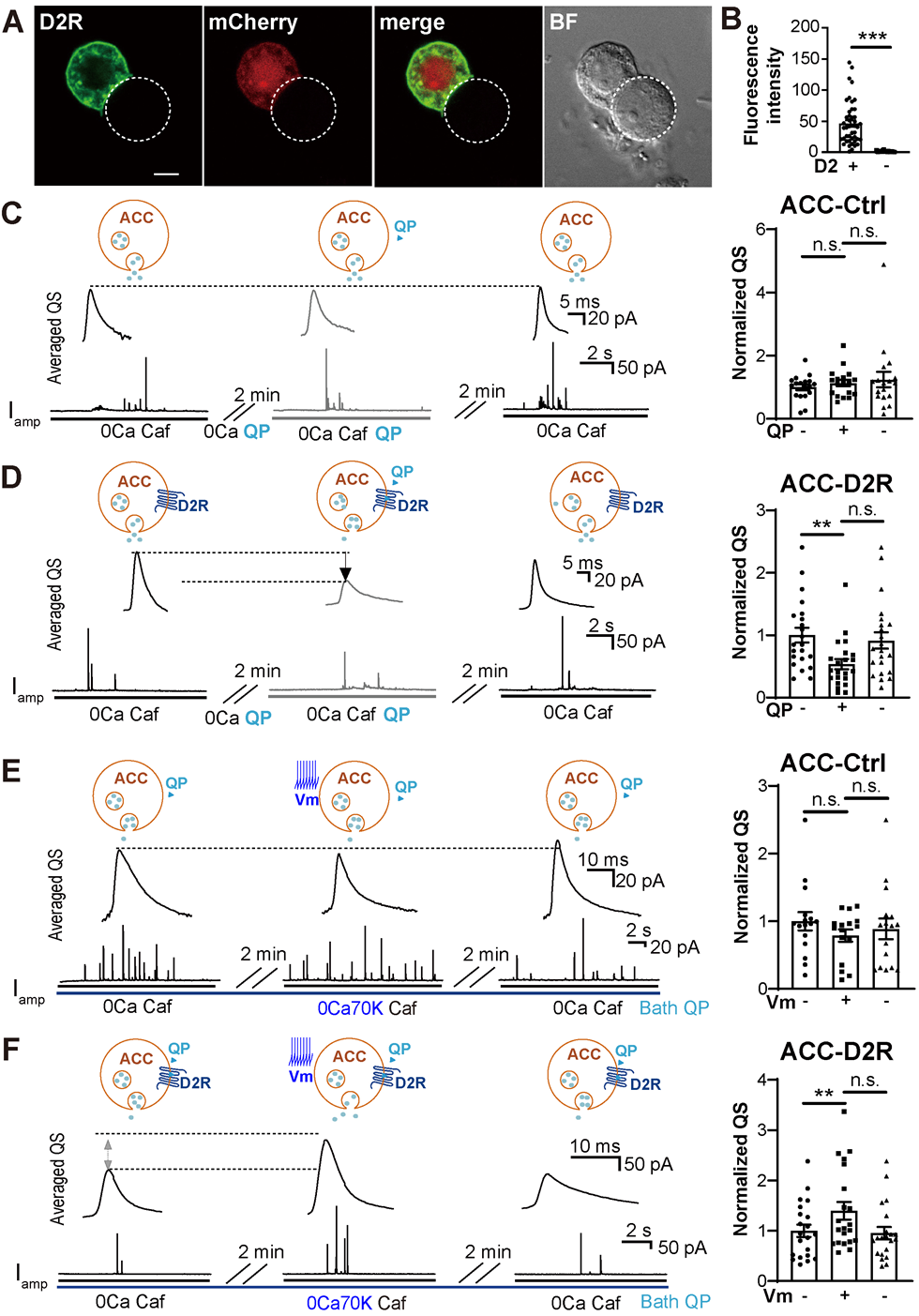
Figure S11 (related to Figure 3). Regulatory effect of the D2R on quantal vesicle release in ACCs**

A) D2R immunostaining in blank and D2R-2A-mCherry-transfected ACCs. A non-fused D2R-2A-mCherry expressing plasmid was used, which express D2R (green, IF with D2R antibody) and mCherry (red) separately. Blank/native ACC has no endogenous D2R expression, while D2R is overexpressed in the D2R-2A-mCherry transfected chromaffin cell (scale bar, 5 μm). B) Statistics of D2R immunostaining in blank and D2R-2A-mCherry-transfected ACCs. (Wilcoxon test, ****p* < 0.001, *n* = 45 cells per group). C) Representative amperometric recordings and statistics of quantal vesicle release evoked by 20 mM caffeine (Caf) before and after QP treatment in a control plasmid GFP-transfected ACC (ACC-Ctrl). QP treatment does not change QS in ACC-Ctrl cells (Friedman test, post hoc Dunn’s multiple comparisons test, *p* > 0.99, n.s., not significant, *n* = 18 cells). D) Representative amperometric recordings and statistics of quantal vesicle release evoked by 20 mM Caf before and after QP treatment in D2R plasmid-transfected ACCs (ACC-D2R). QP treatment significantly inhibits QS in ACC-D2R cells (Friedman test, post hoc Dunn’s multiple comparisons test, ***p* < 0.01, *n* = 22 cells). E) Representative amperometric recordings and statistics of caffeine (Caf, 20 mM) evoked quantal vesicle release from ACC-Ctrl cells bathed in Ca^2+^-free and 2 μM QP-containing extracellular solution (Friedman test, followed by post hoc Dunn’s multiple comparisons test, *p* = 0.43, n.s., not significant, *n* = 16 cells). F) Similar as in (E), except that ACC-D2R cells were used (Friedman test, followed by post hoc Dunn’s multiple comparisons test, n.s., *n* = 21 cells). Data are presented as the mean ± SEM. ****p*< 0.001, ***p* < 0.01, *p* > 0.05, n.s., not significant.


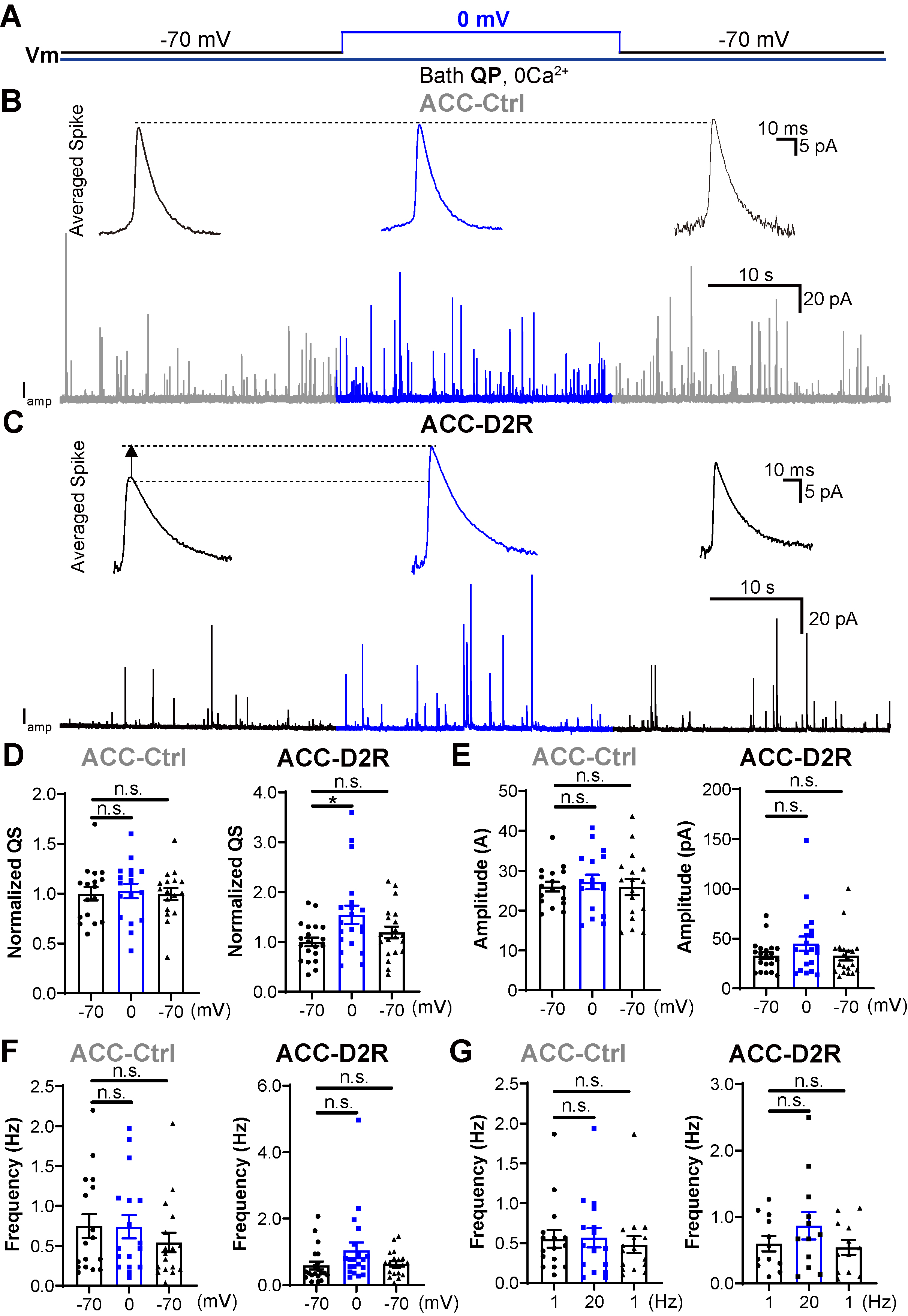
**Figure S12 (related to Figure 3). D2R’s voltage-dependence is validated by depolarization in D2R-overexpressing ACCs.**

A) The diagram shows the stimulation protocol in ACCs. ACCs are bathed in 2 μM QP and 0 Ca^2+^ when quantal vesicle release is triggered by 1 mM intracellular Ca^2+^ in the patch pipette and recorded by a CFE. ACCs are depolarized from –70 mV to 0 mV during quantal vesicle release. B, C) Representative amperometric recordings of quantal vesicle release evoked by the stimulation protocol in panel (A) from ACCs transfected with control plasmid GFP (B, ACC-Ctrl) and D2R plasmid (C, ACC-D2R). The upper traces show the averaged quantal size (QS) corresponding to three periods of stimulation at [-70mV 30 s] (gray or black) *vs* [0 mV 30 s] (blue). D) Statistics of normalized QS. For ACC-Ctrl, QS does not differ between depolarization (0 mV) and resting Vm (–70 mV) (Friedman test, post hoc Dunn’s multiple comparisons test, *p* > 0.99, n.s., not significant, *n* = 17 cells). For ACC-D2R, depolarization significantly increases QS compared to resting Vm (Friedman test, post hoc Dunn’s multiple comparisons test, ****p* < 0.001, *n* = 20 cells). E) Statistics of the quantal release amplitude during -70 mV vs 0 mV stimulation in ACC-Ctrl or ACC-D2R (One-way ANOVA, post hoc Tukey’s multiple comparisons test, *p* > 0.05, *n* = 17 cells for ACC-Ctrl and *n* = 20 cells for ACC-D2R). F) Statistics of the quantal release frequency during -70 mV vs 0 mV stimulation in ACC-Ctrl or ACC-D2R (One-way ANOVA, post hoc Tukey’s multiple comparisons test, *p* > 0.05, *n* = 17 cells for ACC-Ctrl and *n* = 20 cells for ACC-D2R). G) Statistics of the quantal release frequency during 1 Hz vs 20 Hz stimulation in ACCs transfected with control (ACC-Ctrl) or D2R-expressing (ACC-D2R) plasmid (One-way ANOVA, post hoc Tukey’s multiple comparisons test, *p* > 0.05, n.s., not significant, *n* = 16 cells for ACC-Ctrl and *n* = 12 cells for ACC-D2R). Data are presented as the mean ± SEM. ****p* < 0.001, ***p* < 0.01, *p* > 0.05, n.s., not significant.

**
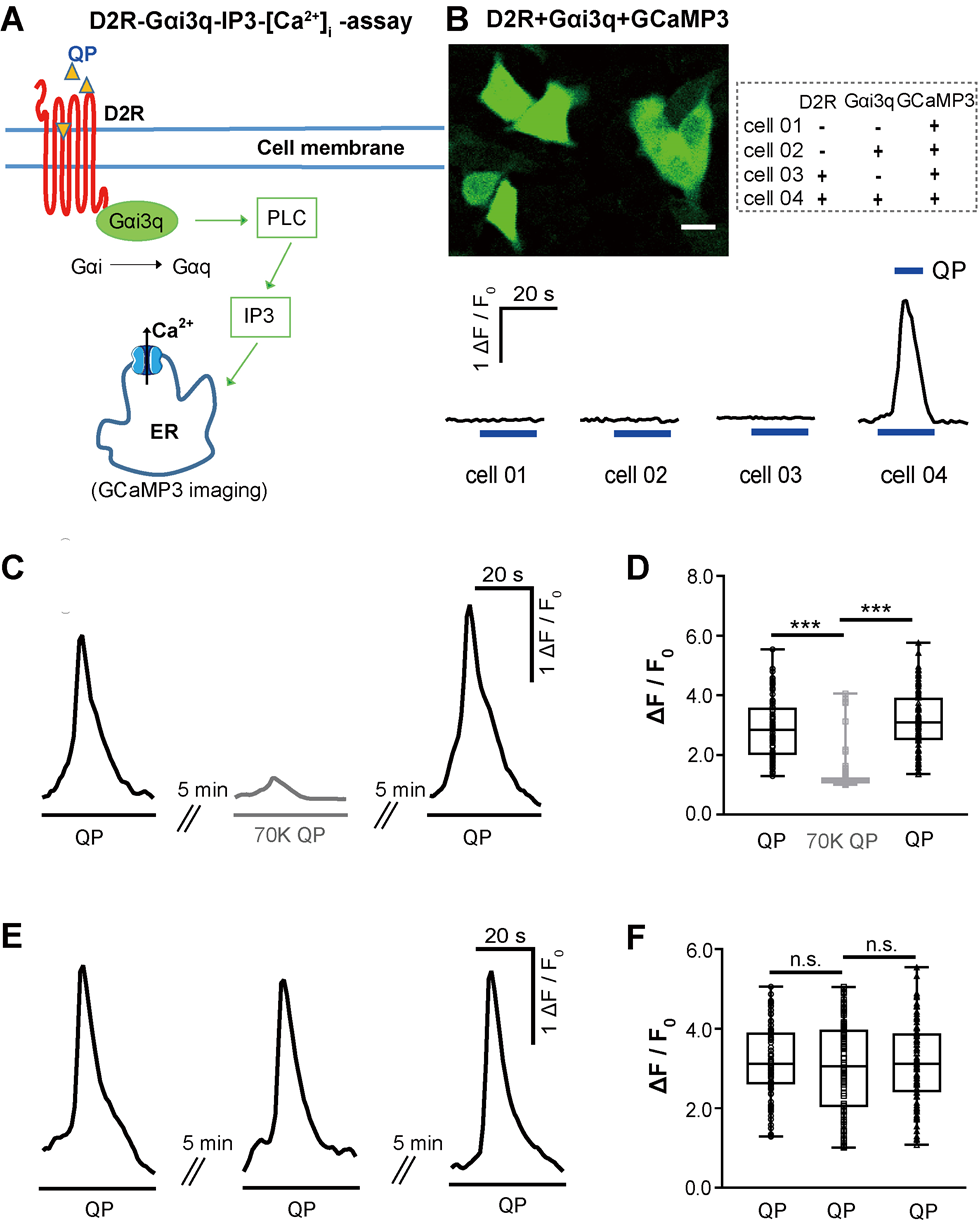
**

**Figure S13 (related to Figure 4). Identification of voltage-dependence of D2R by [Ca^2+^]_i_ imaging.**

A) Cartoon of the D2R-Gαi3q-IP3-[Ca^2+^]_i_ assay. HeLa cells are transfected with the plasmids D2R, GCaMP3, and Gαi3q. The D2R agonist quinpirole (2 μM) activates the D2R-Gαi3q-PLC-IP3 pathway to induce Ca^2+^ release from the ER. The Gαi3q chimera is coupled with Gαi-coupled receptors but transduces signals through the Gαq-mediated PLC-IP3-[Ca^2+^]_i_ mobilization pathway. B) Upper, confocal image of multiple HeLa cells transfected with D2R, Gαi3q, and GCaMP3 plasmids. Lower, QP only triggers [Ca^2+^]_i_ elevation in cell 04 co-expressing three plasmids D2R, GCaMP3, and Gαi3q together, but has no effect on [Ca^2+^]_i_ in cell 01 (only GCaMP3 expressed), cell 02, or cell03 (only two plasmids GCaMP3 and Gαi3q or D2R and GCaMP3 co-expressed). C) Typical traces of [Ca^2+^]_i_ (ΔF/F_0_) triggered by 2 μM QP with or without depolarization induced by 70K. D) Statistics showing that depolarization reversibly decreases ΔF/F_0_ peak, which suggests that D2R is Vm-sensitive (group 1 *vs* group 2, ****p* < 0.001, group 2 *vs* group 3, ****p* < 0.001, Friedman test, post hoc Dunn’s multiple comparisons test, *n* = 95 cells). E, F) Typical traces and statistics of stable [Ca^2+^]_i_ triggered by QP (group 1 *vs* group 2, *p* = 0.52, n.s., not significant, group 2 *vs* group 3, *p* = 0.74, n.s., not significant, Friedman test, post hoc Dunn’s multiple comparisons test, *n* = 90 cells). Data are presented as the mean ± SEM. ****p* < 0.001; *p* > 0.05, n.s., not significant.


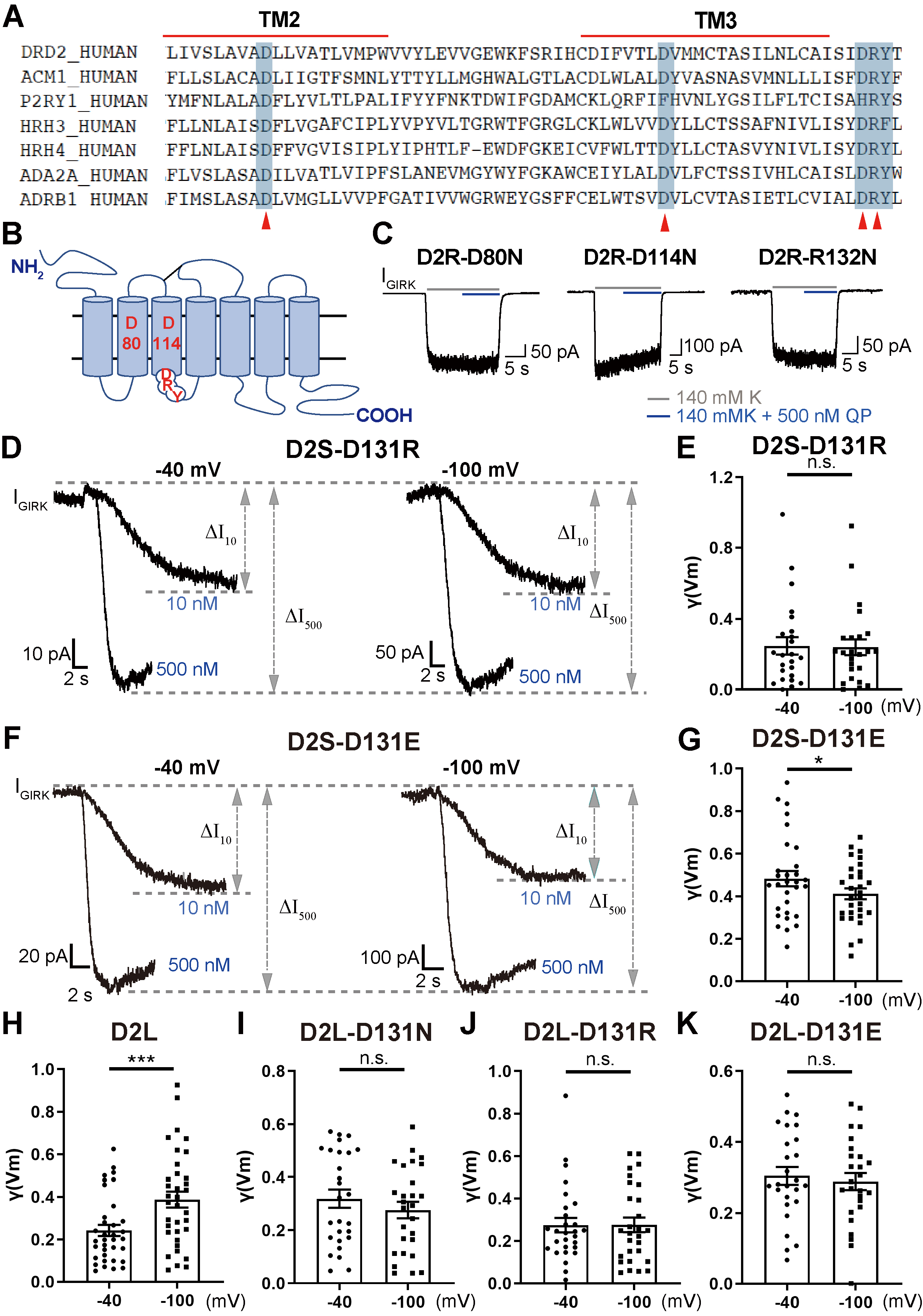
**Figure S14 (related to Figure 4). D2R related mutations alter the voltage sensitivity of D2R compared with D2R-WT using the reconstituted D2R-GIRK system**

A) Alignment of amino-acids of rhodopsin-like GPCRs. Triangles indicate the positions of the charged, transmembrane, and conserved residues: D80, D114, D131, and R132. B) Cartoon showing the D2R topology and positions of D80, D114, D131, and R132. C) D2R mutants D80N, D114N, and R132N all fail to respond to the D2R agonist QP. D) Representative I_GIRK_ recordings in HEK293A cells co-expressing the D2 short isoform (D2S) D131R mutation and GIRK1/4 plasmids. Vm held at –40 mV (left) or –100 mV (right) and I_GIRK_ is triggered by 10 nM and 500 nM QP. E) Statistics of panel (D). For D2S-D131R, the I_GIRK_ ratio γ(Vm) does not differ at –40 mV and –100 mV (Wilcoxon test, *p* = 0.83, n.s., not significant, *n* = 24 cells). F) Similar to panel (D), but in HEK293A cells co-expressing the D2S-D131E mutation and GIRK1/4 plasmids. G) Statistics of panel (F). For D2S-D131E, the I_GIRK_ ratio γ(Vm) is higher at –40 mV than that at –100 mV (paired Student’s t-test, * *p* < 0.05 for D131E, *n* = 30 cells). H-K) Statistics of the I_GIRK_ ratio γ(Vm) in D2 long isoform (D2L) and related mutations. For D2L, the I_GIRK_ ratio γ(Vm) is lower at –40 mV than that at –100 mV, for D2L-D131N/R/E, the I_GIRK_ ratio γ(Vm) does not differ at –40 mV and –100 mV (paired Student’s t-test, for D2L, *n* = 35 cells; for D2L-D131N, *n* = 27 cells; for D2L-D131R, *n* = 28 cells; for D2L-D131E, *n* = 26 cells). Data are presented as the mean ± SEM (E, G, H-K). ****p* < 0.001; **p* < 0.05; *p* > 0.05, n.s., not significant.


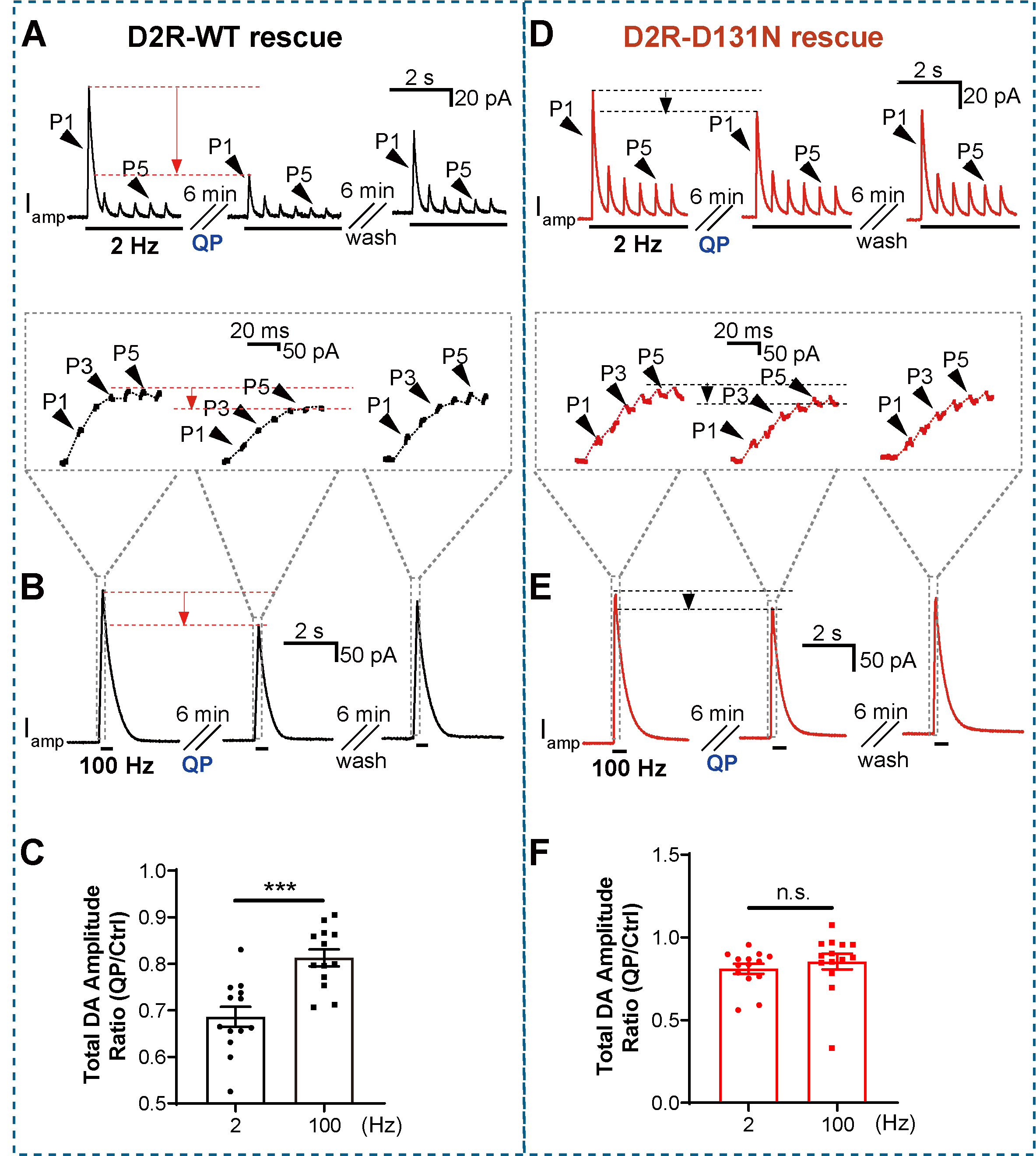


**Figure S15 (related to Figures 6 and 7). D131 is the voltage-sensing site for AP frequency-dependent D2R modulation of DA release in striatal slices**

A, B) Representative traces of evoked DA release before and after treatment with the D2R agonist QP (0.5 μM) with E-stim at [2 Hz, 6 pulses] (A) *vs* [100 Hz, 6 pulses] (B) in adult D2R-KO mice overexpressing D2R-WT virus (D2R-WT rescue). Dashed box, enlargement showing details of DA signals in response to 6-pulse E-stim (P1 to P6). C) Statistics of the effect of QP on total DA release (summary of amplitude I_P1_+I_P2_+…+ I_P6_) at two E-stim frequencies: [2 Hz, 6 P] *vs* [100 Hz, 6 P]. Results show that QP inhibits more DA release at 2 Hz than 100 Hz in D2R-WT rescue mice (Wilcoxon test, ****p*< 0.001, *n* = 13 slices from 4 mice). D, E) Representative traces of evoked DA release before and after D2R agonist QP treatment with E-stim patterns [2 Hz, 6 P] (D) *vs* [100 Hz, 6 P] (E) in adult D2R-KO mice overexpressing D2R-D131N virus (D2R-D131N rescue). Dashed box, enlargement showing details of DA signals in response to 6 pulses of E-stim (P1 to P6). F) Similar to panel (C), but statistics of the QP effect on total DA release in D2R-D131N rescue mice. QP inhibits DA release to the same level at both AP frequencies, indicating that the frequency-dependent D2R modulation of DA release is abolished when the voltage-sensing site D131 is mutated (Wilcoxon test, *p* = 0.17, n.s., not significant, *n* = 14 slices from 5 mice). Data are presented as the mean ± SEM. ****p* < 0.001; *p* > 0.05, n.s., not significant.


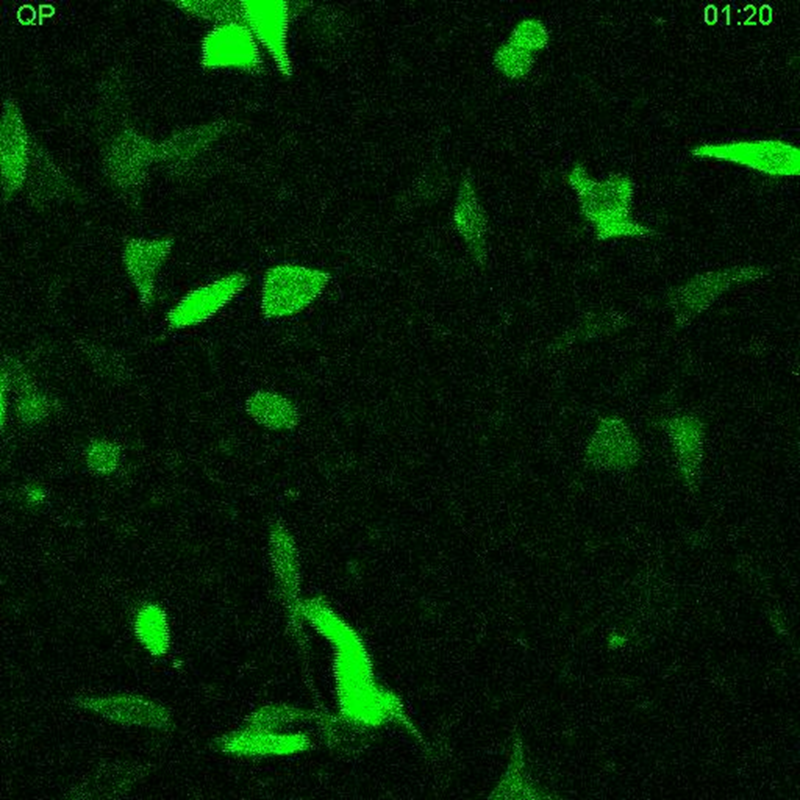


**Movie S1 (related to Figure 4).** In HeLa cells transfected with D2R, Gai3q, and GCaMP3, the D2R agonist quinpirole (2 μM) activates the D2R-Gαi3q-PLC-IP3 signaling pathway (Fig S14) and induces Ca^2+^ release from ER stores. The Ca^2+^ signals were captured by confocal microscopy.


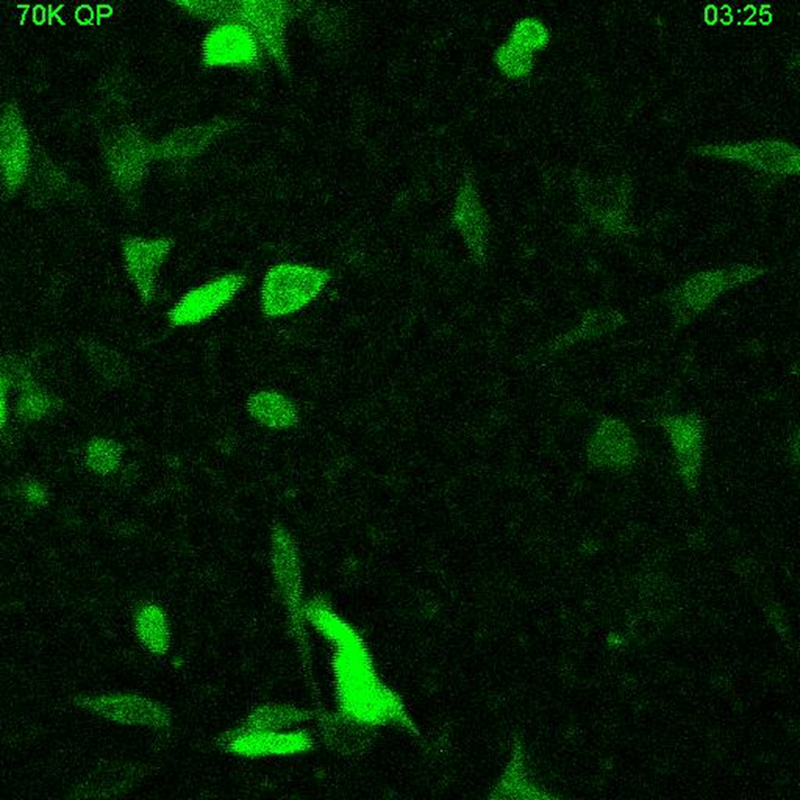


**Movie S2 (related to Figure 4):** As in Movie S1, except with 70 mM KCl depolarization and [Ca^2+^]_i_ is decreased.


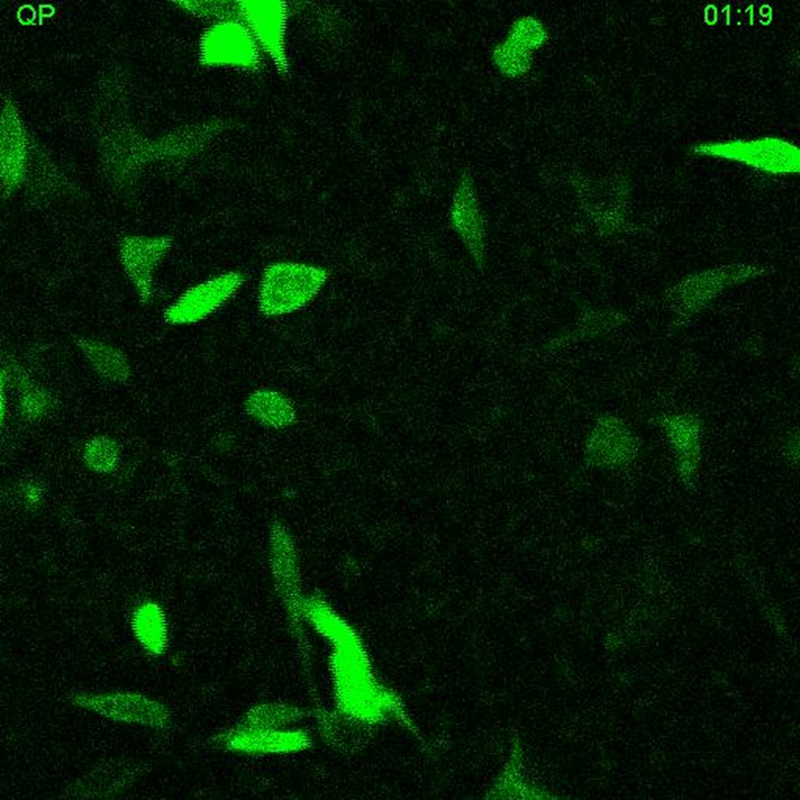


**Movie S3 (related to Figure 4):** As for Movie S2, except without 70 mM KCl depolarization and [Ca^2+^]_i_ is restored. Movie S1–S3 are from the same HeLa cells.
